# Screw pull-out force predictions in porcine radii using efficient nonlinear µFE models including contact and pre-damage

**DOI:** 10.3389/fbioe.2025.1524235

**Published:** 2025-03-24

**Authors:** Pia Stefanek, J. D. Silva-Henao, Victoria Fiedler, A. G. Reisinger, Dieter H. Pahr, Alexander Synek

**Affiliations:** ^1^ Institute of Lightweight Design and Structural Biomechanics, TU Wien, Vienna, Austria; ^2^ Division Biomechanics, Karl Landsteiner University of Health Sciences, Krems, Austria

**Keywords:** micro finite element modeling, bone-screw systems, bone-screw contact, predamage due to screw insertion, efficient materially-nonlinear simulations

## Abstract

Nonlinear micro finite element (µFE) models have become the gold-standard for accurate numerical modeling of bone-screw systems. However, the detailed representation of bone microstructure, along with the inclusion of nonlinear material and contact, and pre-damage due to pre-drilling and screw-insertion, constitute significant computational demands and restrict model sizes. The goal of this study was to evaluate the agreement of screw pull-out predictions of computationally efficient, materially nonlinear µFE models with experimental measurements, taking both contact interface and pre-damage into account in a simplified way. Screw pull-out force was experimentally measured in ten porcine radius biopsies, and specimen-specific, voxel-based µFE models were created mimicking the experimental setup. µFE models with three levels of modeling details were compared: Fully bonded interface without pre-damage (FB), simplified contact interface without pre-damage (TED-M), and simplified contact interface with pre-damage (TED-M + P). In the TED-M + P models, the influence of pre-damage parameters (damage zone radial thickness and amount of damage) was assessed and optimal parameters were identified. The results revealed that pre-damage parameters highly impact the pull-out force predictions, and that the optimal parameters are ambiguous and dependent on the chosen bone material properties. Although all µFE models demonstrated high correlations with experimental data (*R* ^2^ > 0.85), they differed in their 1:1 correspondence. The FB and TED-M models overestimated maximum force predictions (mean absolute percentage error (MAPE) > 52%), while the TED-M + P model with optimized pre-damage parameters improved the predictions (MAPE <17%). In conclusion, screw pull-out forces predicted with computationally efficient, materially nonlinear µFE models showed strong correlations with experimental measurements. To achieve quantitatively accurate results, precise coordination of contact modeling, pre-damage representation, and material properties is essential.

## 1 Introduction

While experimental testing with cadaver bones remains the gold standard in bone-implant research, finite element (FE) analysis offers significant advantages. Experiments are time-consuming, costly, and require scarce human or animal tissue. In contrast, once an FE model is created and validated, parameters can be easily modified, allowing for efficient testing of various implant configurations on the same subject. This makes FE analysis ideal for systematic optimization studies. FE models provide a deeper understanding of local stress and strain, helping to identify potential weaknesses and failure points (Lewis et al., 2021; Synek et al., 2015; Taylor and Prendergast, 2015). Micro-FE (µFE) models, based on high-resolution CT images, are currently considered the benchmark for bone-screw modeling. They capture the local bone microstructure, including trabecular networks and screw geometry, which is crucial for accurately predicting mechanical behavior and anchorage quality (Marcián et al., 2021; Wirth et al., 2011; Wirth et al., 2012). Nonetheless, the detailed representation of microstructure increases µFE model sizes and computational demands. Moreover, recent studies highlight the need to incorporate nonlinearities in bone-screw simulations, as both bone failure and bone-screw contact interactions are nonlinear processes (Ovesy et al., 2022; Panagiotopoulou et al., 2021; Zhou et al., 2024). However, including these nonlinearities, increases model complexity and computational requirements even more.

One possibility to deal with high computational demands in µFE are specialized solvers developed to solve large-scale problems with several millions of elements (e.g., FEAP (Taylor, 2020), Faim [Numerics88 Solutions Ltd., https://bonelab.github.io/n88/index.html), ParOSol (Flaig and Arbenz, 2012), ParOSol-NL (Stipsitz et al., 2020)]. These solvers are highly efficient for solving large-scale problems, but in turn often only support linear-elastic or simplified nonlinear material laws. As they generally lack the ability to include nonlinear contact mechanics, some studies have proposed simplified contact models to overcome these limitations, while maintaining computational efficiency (Stefanek et al., 2024; Steiner et al., 2017). While the relevance of interface modeling in bone-screw µFE simulations has already been demonstrated (Stefanek et al., 2024), modeling of peri-implant bone damage due to pre-drilling and screw insertion may be even more critical for accurate predictions (Steiner et al., 2017; Zhou et al., 2024). Various studies have reported that the screw insertion causes damage in the surrounding bone (Lee and Baek, 2010; Steiner et al., 2016; Wang et al., 2012; Warreth et al., 2009). Steiner et al. (2016) localized and quantified the screw insertion related pre-damage by comparing µCT scans of human femoral bone before and after screw insertion. They found that the damaged region depends on the screw thread depth and can extend up to a radial distance of 0.9 mm, with the most significant damage occurring within a distance of 0.3 mm.

However, in general it remains unclear how to define the radial thickness of the damage zone in a µFE simulation and how to model the compromised mechanical properties inside the damage zone. Consequently, literature research reveals a variety of pre-damage modeling approaches. Ovesy et al. (2019) and Zhou et al. (2024) conducted nonlinear simulations incorporating the screw insertion process, but this method is computationally intensive and feasible only for small models. To maintain computational efficiency, other studies used linear simulations and defined damage zones around the screw with a uniformly reduced elastic modulus (Chevalier et al., 2018; Steiner et al., 2017; Torcasio et al., 2012). Damage zones were selected with radial thicknesses between 0.16 mm (Torcasio et al., 2012) and 0.9 mm (Steiner et al., 2017) around the screws. Inside these zones, the bone elastic modulus was reduced between 17% (Chevalier et al., 2018) and 99.5% (Torcasio et al., 2012). As all studies used bones from different species (human, rat) and anatomical locations (spine, femur, hind limb), they selected different elastic moduli for undamaged bone. Hence, this wide range of damage estimation could also result from variations in the selection of material properties. To the author’s knowledge, no study has yet tried to implement this simplified pre-damage modeling approach in computationally efficient µFE simulations with nonlinear material.

The objective of this study was to assess the correlation between screw pull-out predictions from computationally efficient, materially nonlinear µFE models and experimental measurements, while considering both contact interface and pre-damage. In a first step, the parameters of a simplified pre-damage model were identified dependent on the elastic modulus selection. In a second step, the predicted maximum force of different computationally efficient µFE models was compared to experiments and damage distributions were evaluated.

## 2 Materials and methods

**FIGURE 1:**
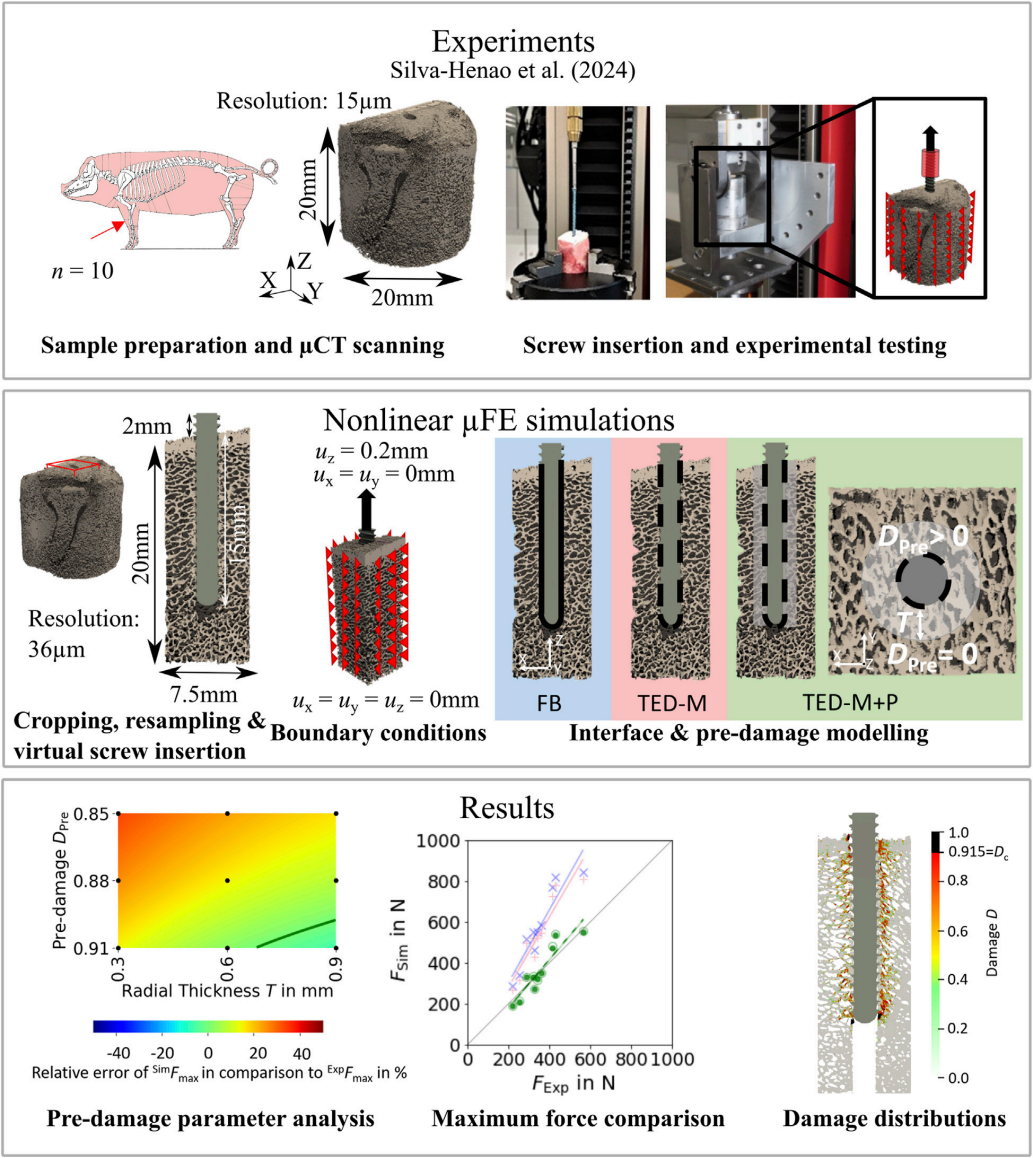
Study outline. Predicted pull-out force of nonlinear µFE simulations of bone-screw models was compared to experiments. The simulations included both a simplified contact interface and a simplified pre-damage model. The photographs from the experimental procedure were taken from Silva-Henao et al. (2024).

Figure 1 provides an overview of this study. The experimental part was conducted by Silva-Henao et al. (2024). Screws were inserted into porcine distal radius biopsies after pre-drilling, and experiments were conducted to assess maximum pull-out force. µCT scans of the pre-drilled samples were cropped and used to generate nonlinear µFE models that replicated the experiments. After determining optimal pre-damage parameters for different elastic moduli, three different interface and pre-damage modeling combinations were implemented, and the predicted maximum force was compared to experimental measurements. Additionally, damage distributions at maximum force were evaluated.

### 2.1 Experimental data

The study is based on the experimental data of Silva-Henao et al. (2024), where ten porcine distal radii were selected for pull-out experiments (see Figure 1). A conventional drill-press with a modified core driller was used to extract a cylindrical sample (20 mm in diameter and height) with a centered 2 mm pilot hole from each bone. Following the implantation procedure described in Ovesy et al. (2022), a universal mechanical testing machine (ZwickiLine Z2.5, ZwickRoell GmbH & Co. KG, Ulm, Germany) was used to implant a locking screw (outer diameter: 2.5mm; titanium alloy TiAl6V4; A-5750, Medartis Inc., Basel, Switzerland). The screw was inserted mono-cortically to a depth of 15 mm. Tensile force-controlled loading (loading rate: 50N/s) was applied using a custom-designed testing apparatus. The samples were laterally fixed in a sample holder, while cyclic loading was applied to the screw via a clamp positioned in 2 mm distance to the bone. The loading started with a pre-conditioning phase including 20 loading cycles between 0N and 15N which was followed by a pause of 1s. Afterwards, a cyclic overloading phase followed, where the load amplitude was increased by 1N per cycle while maintaining a minimum load of 15N. The loading was applied until the screw was entirely pulled out of the bone samples.

### 2.2 Image processing

µCT images with a resolution of 15 µm were acquired from the unloaded, pre-drilled bone samples using a SkyScan1173 µCT scanner (Bruker, Bilerica, United States) (90 kV source voltage, 60 μA source current, 1250 ms exposure time, 1 mm aluminum filter). In order to reduce image noise, the μCT images were smoothed using a Gaussian filter (σ = 1; kernel size = 2 × 2 × 2). The samples were aligned along the pre-drilled screw axis, and cuboids with a square cross section (7.5 mm side length) were cropped from the bone center (see Figure 1). The cuboid size was determined following Ovesy et al. (2019), Ovesy et al. (2022) to minimize simulation times while ensuring to fully capture bone damage around the screw. Single-level thresholding was applied to binarize the images. A µCT image (resolution: 14.8 μm; SkyScan1173, Bruker, Bilerica, United States) of the same locking screw that was used for the pull-out experiments, described in section 2.1, was taken from a previous study (Synek et al., 2023) and cropped to a length of 17 mm. Bone and screw images were resampled to a resolution of 36 μm, as the later applied material model of Stipsitz et al. (2020) (see Section 2.3) was developed for resolutions of this magnitude. The screw was virtually inserted to a depth of 15 mm into the center of the segmented bone images to mimic the experimental conditions. The samples had a bone volume fraction range of 18.2%–38.3% and their mean cortical thickness varied between 237 µm and 1124 µm. All image processing steps and morphometric evaluations were performed with Medtool 4.5 (Dr. Pahr Ingenieurs e.U., Pfaffstätten, Austria).

### 2.3 Mesh, material and boundary conditions

The segmented bone images with the virtually inserted screw were used as geometrical input to generate µFE models. Each voxel was converted into eight-noded hexahedral elements (side length: 36 µm). The number of elements varied between 5.8 and 10.3 million. Material properties were assumed to be homogeneous and isotropic. For the bone material, a nonlinear damage-based material model (Stipsitz et al., 2020; Stipsitz et al., 2021) especially developed for efficient nonlinear µFE analysis was selected. The model consists of a linear-elastic region, a damaged region, and a failure region. In the linear-elastic region, an elastic modulus E= 4.6 GPa and a Poisson’s ratio of ν = 0.3 were selected. As material parameters proposed by Stipsitz et al. (2020), Stipsitz et al. (2021) (*E* = 10 GPa) were identified for human rather than porcine bone, the elastic modulus was taken from Costa et al. (2017), who found the best fit between µFE predicted and experimental axial forces on a porcine bone sample using *E* = 4.6 GPa. The transition from the linear to the nonlinear, damaged regime was modeled using an isotropic, quadric damage onset surface (shape parameter ζ = 0.3) which differentiates between tension and compression. Since Morgan and Keaveny (2001) showed that yield strains remain relatively constant across species, damage onset strains in tension and compression were taken from Stipsitz et al. (2020), Stipsitz et al. (2021) and kept constant (damage onset strain in tension $\varepsilon^{+}=$ 0.0068; damage onset strain in compression $\varepsilon^{-}=$ 0.0089), while damage onset stresses were scaled according to the selected elastic modulus (Ovesy et al., 2019; Panagiotopoulou et al., 2021). In the damaged region, isotropic material hardening [ $E_{H}=$ 0.05 $E_{0}$ ) (Stipsitz et al. (2020), Stipsitz et al. (2021)] was included, and material degradation, found by back-projection of the current stress state on the damage onset surface, was modeled via local stiffness reduction according to observed damage levels. When the critical damage threshold $D_{c}$ = 0.915 (Stipsitz et al. (2020), Stipsitz et al. (2021)) was exceeded, local failure was modeled by reducing the elastic modulus to a residual value close to zero. For the titanium alloy screw, linear-elastic material properties, with an elastic modulus of E= 115 GPa and a Poisson’s ratio of ν = 0.3 (Synek et al., 2023) were assumed.

The boundary conditions were selected to mimic the experimental conditions (see Figure 1). The nodes located on the four lateral sides of the bone were fully constrained. At the screw top, a displacement of 0.2 mm was applied along the screw axis, while the nodes were constrained in all other directions. The displacement value of 0.2 mm was selected since it was enough to observe a drop in the force-displacement curve in all simulations. The displacements obtained from the simulations could not be directly compared to the experimentally measured displacements, as only crosshead displacements were measured in the experiments. Additionally, cropping the specimen geometry in the µFE models influenced the displacement results.

All µFE models were generated with the software Medtool 4.5 (Dr. Pahr Ingenieurs e.U., Pfaffstätten, Austria).

### 2.4 Interface modeling

Three types of µFE models with different interface and pre-damage combinations (see Table 1) were compared: a fully-bonded interface without pre-damage (FB), a modified tensionally-strained element deletion (TED-M) (Stefanek et al., 2024; Steiner et al., 2017) interface without pre-damage, and a TED-M interface with pre-damage (TED-M + P). The fully-bonded interface assumes perfect bonding at the bone-screw interface nodes. TED-M represents a modification of the tensionally-strained element deletion (TED) interface model introduced by Steiner et al. (2017), which imitates contact in a simplified way. It is based on a preliminary linear-elastic simulation (“pre”-simulation) with a fully-bonded bone-screw interface. Interface elements under positive (tensional) volumetric strain are removed, assuming no tensile stress transfer at the bone-screw interface. Elements under negative (compressional) volumetric strain are retained, as they contribute to stress transfer between bone and screw. Finally, the actual simulation with the updated interface is conducted. Stefanek et al. (2024) extended the model of Steiner et al. (2017) to TED-M which enhances the accuracy of maximum force predictions. In an attempt to better account for occurring contact area changes throughout the simulation process, TED-M slightly expands the contact area found with TED by reincluding neighboring interface elements of contact elements in the contact area.

**TABLE 1:** Description of all three compared interface and pre-damage combinations.

Name	FB	TED-M	TED-M + P	
Schematic illustration	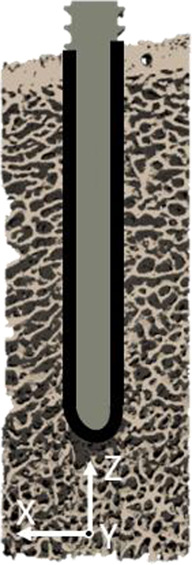	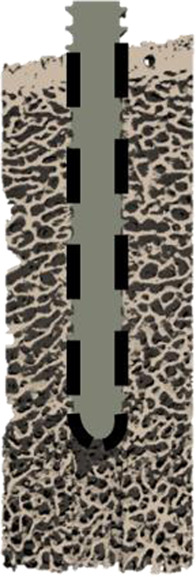	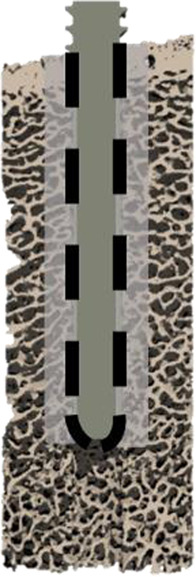	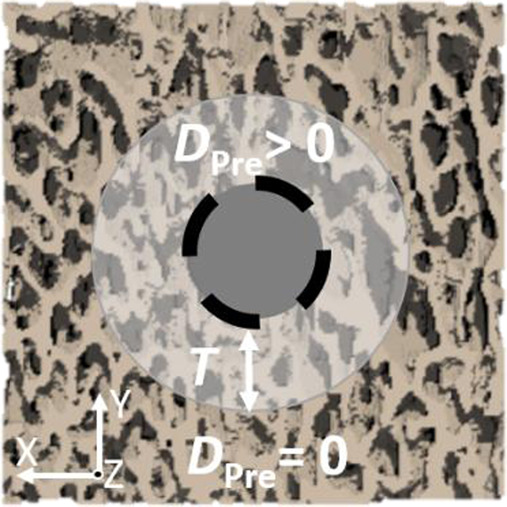
Interface	Fully-bonded	Modified tensionally-strained element deletion^a^	Modified tensionally-strained element deletion^a^	
Pre-damage	None	None	Pre-damage value *D*_Pre_ > 0 in cylindrical zone around screw with radial thickness *T*	

^a^ Stefanek et al. (2024), Steiner et al. (2017).

The fully-bonded (FB) interface assumes bonding between bone and screw, while the modified tensionally-strained element deletion (TED-M) interface deletes selected elements at the interface to better replicate bone-screw contact. The TED-M interface is combined with a simplified pre-damage model. A cylindrical region with a radial thickness *T* is selected, where a pre-damage value *D*_Pre_ > 0 is assigned.

### 2.5 Pre-damage modeling

Pre-damage was modeled by defining a cylindrical damage zone with a radial thickness *T*, where a pre-damage value of bone $D_{\text{Pre}}$ ( $\left.0<D_{\text{Pre}}<D_{c}\right)$ was set (see Figure 1). This approach is slightly different to other simplified pre-damage models reported so far, where the pre-damage was defined as elastic modulus reduction in linear elastic µFE models. Bone-screw research done so far could not provide conclusive guidelines on how to set the pre-damage value ( $D_{\text{Pre}}$ ) and the thickness of the pre-damage zone. Hence, the influence of these parameters on the maximum force was investigated. As we hypothesized that the selection of material properties also affects maximum pull-out force, the influence of reducing and increasing the elastic modulus was also investigated.

To test the influence of pre-damage parameters on maximum pull-out force, 27 different combinations of pre-damage value ( $D_{Pre}$ = 0.85, 0.88, 0.91), radial thickness ( T= 0.3mm, 0.6mm, 0.9 mm) and elastic modulus (*E*_red_ = 3.6GPa, *E* = 4.6GPa, *E*_inc_ = 5.6 GPa) were evaluated for each specimen. The parameter ranges of $D_{Pre}$ and *T* were set following observations in literature (Steiner et al., 2017) and considering the requirements of the used material law ( $D_{\text{Pre}}$ < $D_{c}$ ).

To visualize the error in the µFE predicted maximum pull-out force for each parameter combination, heat maps (cubic interpolation) were generated, covering the parameter space. The error between µFE models and experiments was defined as $\left(\;{\text{Sim}}_{F_{\max}}-{\mathrm{Exp}}_{F_{\max}}/{\mathrm{Exp}}_{F_{\max}}\right)$ . Isolines were evaluated to indicate parameter combinations with a relative error of zero. In cases where no parameter combination led to an error of zero, the parameter combination with lowest relative error was indicated.

To identify optimal per-damage parameters for all specimens, a mean heat map was created by calculating the mean relative error of all specimens for the nine parameter combinations and each elastic modulus. From the isoline of the mean heat map, two parameter sets were extracted for each elastic modulus: one with minimal radial thickness (Min. *T*) and one with minimal damage (Min. *D*_Pre_). These parameter combinations were then used to perform µFE simulations for all specimens, and to compare the µFE predictions with the experimental measurements.

### 2.6 Simulation

All µFE models were solved with ParOSol-NL (Flaig and Arbenz, 2012; Stipsitz et al., 2020) using up to 126 cores on a dual AMD EPYC 7763 64-core processor with 1 TB RAM. Simulations were performed until a drop of force of at least 15N was observed.

### 2.7 Comparison between µFE and experiments

Maximum force values of all µFE simulations (FB, TED-M, and TED-M + P) were compared to experimental results using linear regressions. The following parameters were computed: slope, intercept, coefficient of determination (*R* ^2^), concordance correlation coefficient (CCC), mean absolute percentage error (MAPE), and root mean squared error (RMSE). Damage distributions were evaluated, visualized, and compared for all µFE simulations at maximum force.

All statistical evaluations were conducted with Python 3.8 (https://www.python.org/) and the included library SciPy (Virtanen et al., 2020). The figures showing the damage distribution were generated with the software ParaView (https://www.paraview.org/).

## 3 Results

### 3.1 Influence of radial thickness, pre-damage value and elastic modulus

**FIGURE 2:**
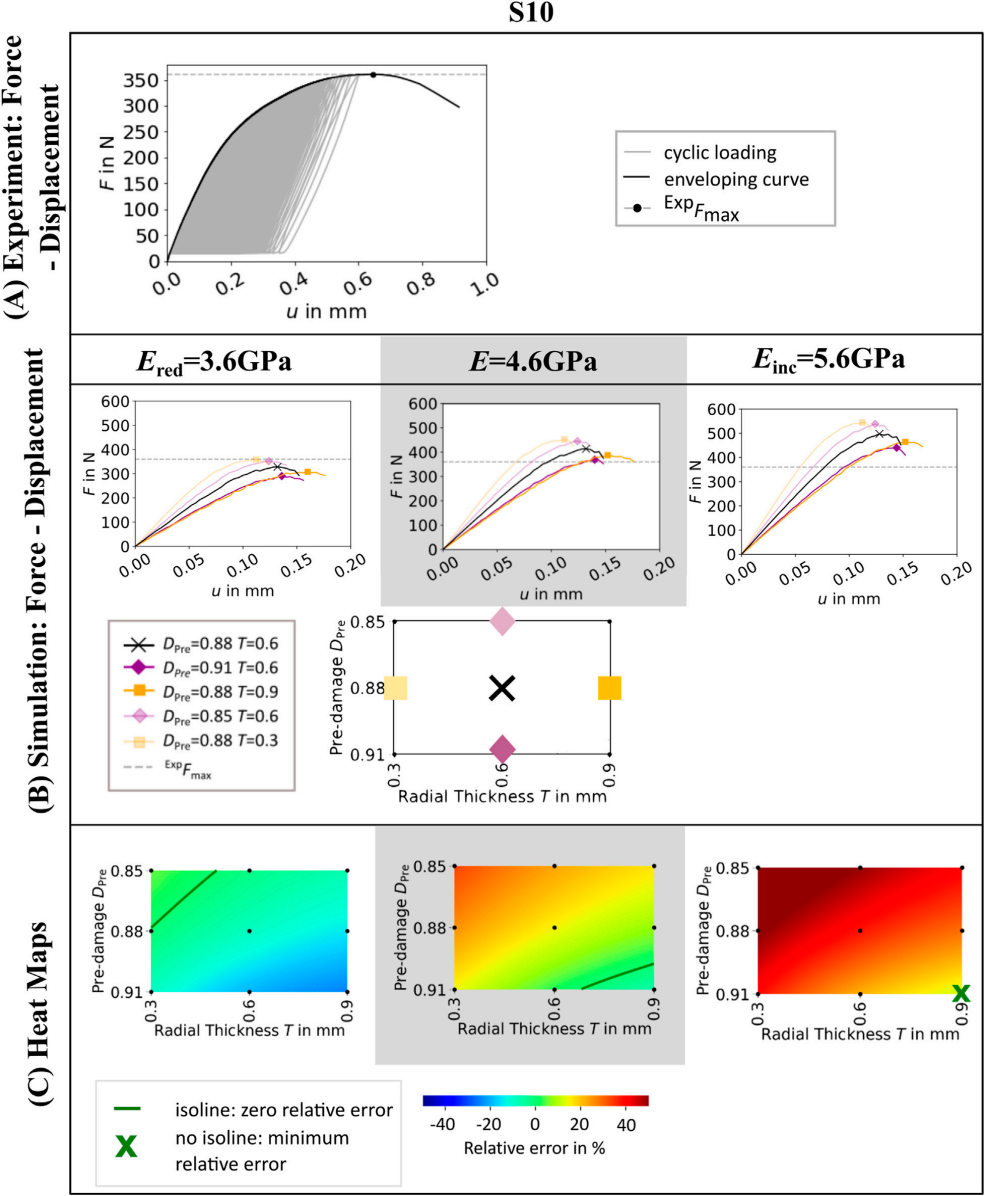
Experimental force-displacement curve **(A)**, simulated force displacement curves for different values of radial thickness *T* and pre-damage value *D*_Pre_ **(B)** and heat maps showing the relative error in maximum force **(C)** of one representative specimen (S10). Simulated force-displacement curves and heat maps are shown for three different elastic moduli of bone material *E*_red_ = 3.6GPa, *E* = 4.6GPa, and *E*_inc_ = 5.6 GPa. The simulated force-displacement curves **(B)** show a selection of five parameter combinations of *T* and *D*_Pre_. In the heat maps **(C)**, green isolines mark the parameter combinations of pre-damage *D*_Pre_ and radial thickness of damage zone *T*, where the relative error in maximum force between simulation and experiment is zero. In case that no parameter combination can be found that leads to zero relative error, the parameter combination where the relative error is minimal is marked by a green cross.

As expected, µFE simulations with lower elastic modulus led to lower maximum force predictions, and *vice versa* (see Figure 2B; Supplementary Material). Both higher pre-damage and larger radial thickness of the pre-damage zone led to lower maximum force predictions. The results differed for each specimen (see Supplementary Material), but tendentially an elastic modulus of *E*_red_ = 3.6 GPa led to underestimation of experimental maximum force, whereas an elastic modulus of *E*_inc_ = 5.6 GPa led to overestimations.

The influence of the chosen elastic modulus and pre-damage parameters on the relative error is also visualized in the heat maps (see Figure 2C; Supplementary Material). The isolines in the heatmap indicated a variety of parameter combinations leading to a relative error of zero percent, depending on the selected elastic modulus (see Figure 2C) and specimen (Supplementary Material). All zero-error isolines showed a similar relation of pre-damage value and radial thickness of the pre-damage zone. Setting the pre-damage to a higher value required smaller radial thickness to achieve zero maximum force error and *vice versa*.

**FIGURE 3:**
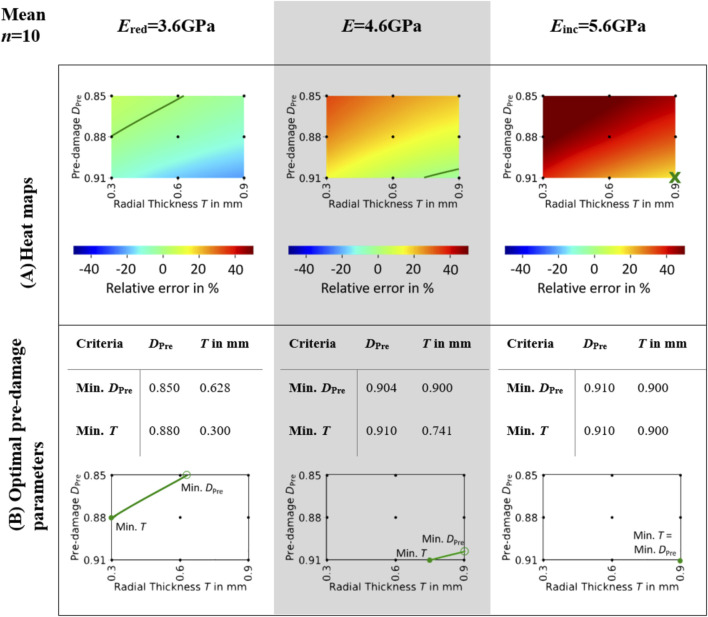
Mean heat maps **(A)** and optimal pre-damage parameters **(B)**. The heat maps **(A)** illustrate the mean relative error across all specimens for three different elastic moduli of bone material: *E*_red_ = 3.6GPa, *E* = 4.6GPa, and *E*_inc_ = 5.6 GPa. Isolines indicate the parameter combinations of pre-damage *D*_*Pre*_ and radial thickness of damage zone *T* where the relative error in maximum force between simulation and experiment is zero. The optimal pre-damage parameters based on the minimal pre-damage (Min. *D*_Pre_) and the minimal radial thickness criteria (Min. *T*) are summarized in **(B)**. The unfilled green markers ‘o’ denote the points where the isoline parameters are evaluated based on the minimum pre-damage criteria, whereas the filled green markers ‘•’ denote the points where the isoline parameters are evaluated based on the minimum radial thickness criteria.

Mean heat maps (see Figure 3A) showed isolines for *E*_red_ = 3.6 GPa and *E* = 4.6GPa, while for *E*_inc_ = 5.6 GPa none of the investigated pre-damage parameter combinations led to a mean error of zero. Hence, the optimal pre-damage parameter combination included both the highest evaluated pre-damage value 0.91 and the highest evaluated radial thickness 0.9 mm. The optimal radial thickness as well as the optimal pre-damage value were lower for *E*_red_ = 3.6 GPa than for *E* = 4.6 GPa for both evaluated criteria Min. *D*_Pre_ and Min. *T* (see Figure 3B). While the optimal pre-damage value ranged between 0.85 and 0.88 for *E*_red_ = 3.6GPa, it needed to be increased between 0.904 and 0.91 to reach best outcomes for *E* = 4.6 GPa. Radial thickness was evaluated to be ideal between 0.3 and 0.628 mm for *E*_red_ = 3.6 GPa and between 0.741 and 0.9 mm for *E* = 4.6 GPa.

### 3.2 Comparison of maximum forces

**FIGURE 4:**
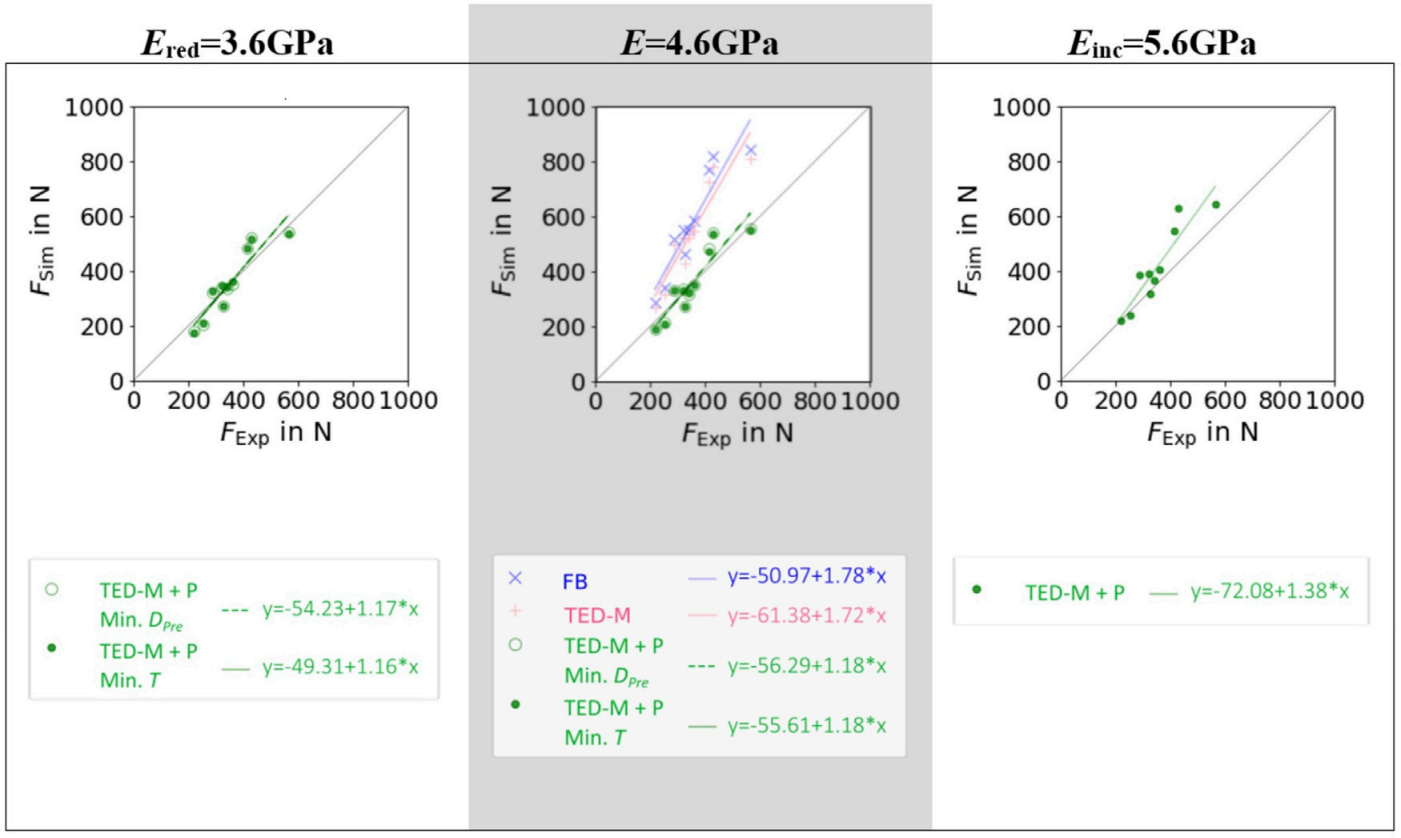
Linear regressions between µFE predicted (*F*_Sim_) and experimentally measured (*F*_Exp_) maximum force for three different elastic moduli of bone material *E*_red_ = 3.6GPa, *E* = 4.6GPa, and *E*_inc_ = 5.6 GPa and three different pre-damage and interface combinations: a fully-bonded interface without pre-damage (FB), a simplified contact model (Stefanek et al., 2024; Steiner et al., 2017) without pre-damage (TED-M), and the same contact model with pre-damage (TED-M + P). Optimal pre-damage parameters were selected based on two criteria defined in Figure 3: minimal pre-damage (Min. *D*_Pre_) and minimal radial thickness criteria (Min. *T*).

Both models without pre-damage implementation (FB, TED-M) showed high correlations (*R* ^2^ > 0.86) to experiments but failed to predict a good 1:1 fit, as confirmed by high RMSE (>211N) and MAPE (>51%) and low CCC (<0.43) (see Figure 4; Table 2). Models with optimal pre-damage implementations (TED-M + P) according to Figure 3B, achieved comparable *R* ^2^ correlations (>0.85) but improved the 1:1 fit to experiments for *E*_red_ = 3.6 GPa and *E* = 4.6 GPa (RMSE<50N; MAPE<12%; CCC>0.89). Only minor differences were observed between *E*_red_ = 3.6 GPa and *E* = 4.6 GPa as well as between different criteria for optimal pre-damage parameter selection (Min. *T* or Min. *D*_Pre_). For *E*_inc_ = 5.6GPa, TED-M + P improved the maximum force predictions in comparison to the models without pre-damage. However, TED-M + P with *E*_inc_ = 5.6 GPa showed inferior results in comparison to the TED-M + P models with *E*_red_ = 3.6 GPa and *E* = 4.6 GPa.

**TABLE 2:** Comparison of linear regressions from Figure 4 using different error metrics to evaluate the goodness of fit.

		RMSE in N	MAPE in %	CCC	*R* ^2^
*E*_red_ = 3.6 GPa	TED-M + P Min. *D*_Pre_	46.74	11.55	0.905	0.864
	TED-M + P Min. *T*	**46.27**	11.60	**0.906**	0.863
*E* = 4.6 GPa	FB	242.58	61.20	0.371	**0.866**
	TED-M	211.18	51.93	0.429	0.865
	TED-M + P Min. *D*_Pre_	49.91	11.68	0.895	0.855
	TED-M + P Min. *T*	47.26	**11.17**	0.904	0.865
*E*_inc_ = 5.6 GPa	TED-M + P	89.26	17.37	0.7658	0.860

Note: root mean squared error (RMSE), mean absolute percentage error (MAPE), concordance correlation coefficient (CCC) and coefficient of determination (*R* ^2^).

For each metric, the model showing the best results (lowest error or highest correlation) was formatted as bold text.

### 3.3 Comparison of damage distributions

**FIGURE 5:**
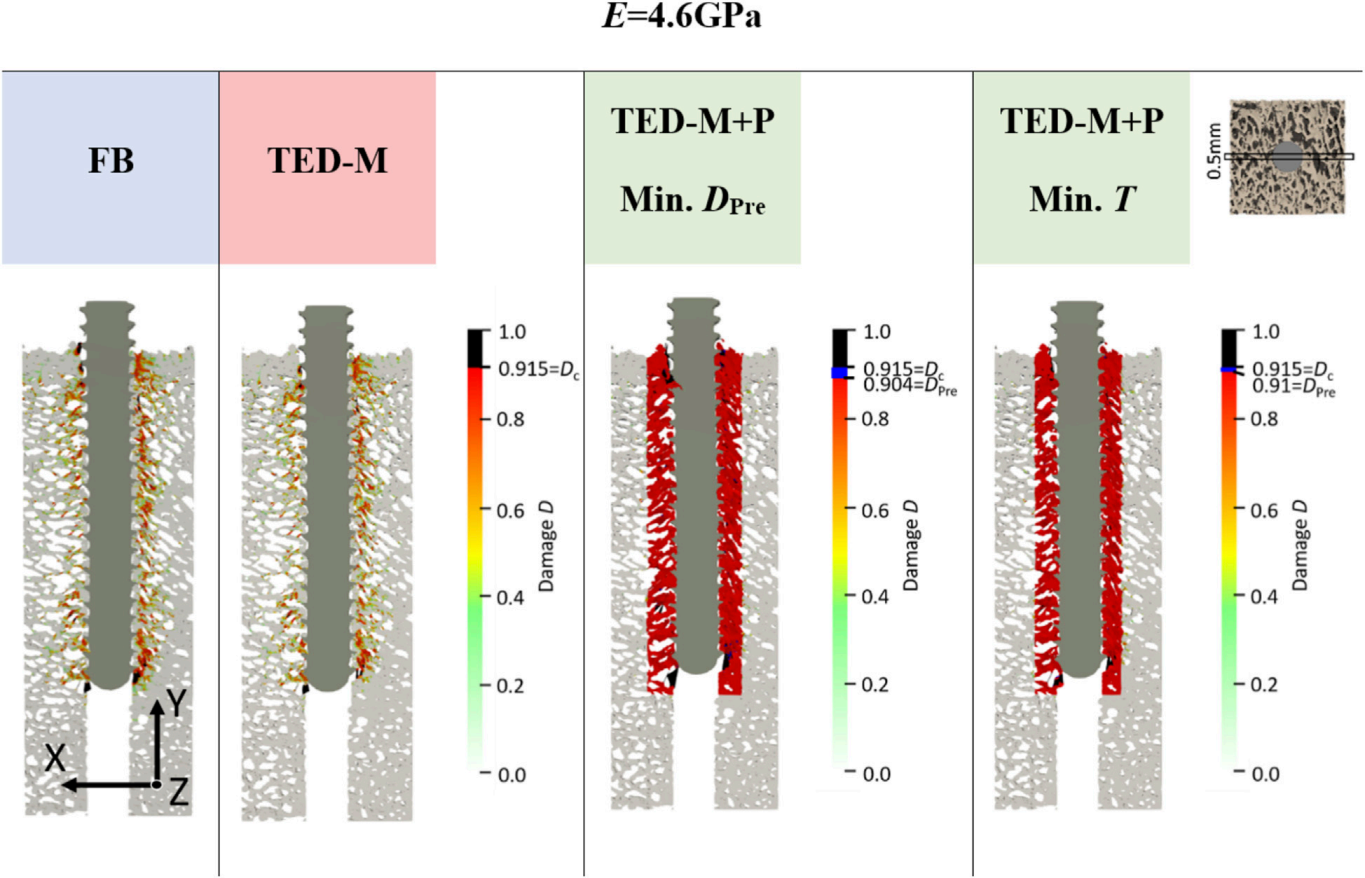
Damage at maximum force of one representative specimen (S10) for *E* = 4.6 GPa and for all interface and pre-damage combinations: a fully-bonded interface without pre-damage (FB) and a simplified interface method (Stefanek et al., 2024; Steiner et al., 2017) without pre-damage (TED-M) and with pre-damage (TED-M + P). A displacement scaling factor of 5 was used, and optimal pre-damage parameters were selected based on two criteria defined in Figure 3: minimal pre-damage (Min. *D*_Pre_) and minimal radial thickness criteria (Min. *T*). For the TED-M + P, the color bar includes blue regions between the pre-damage value *D*_Pre_ in the damage zone and the critical damage *D*_c_.

**FIGURE 6:**
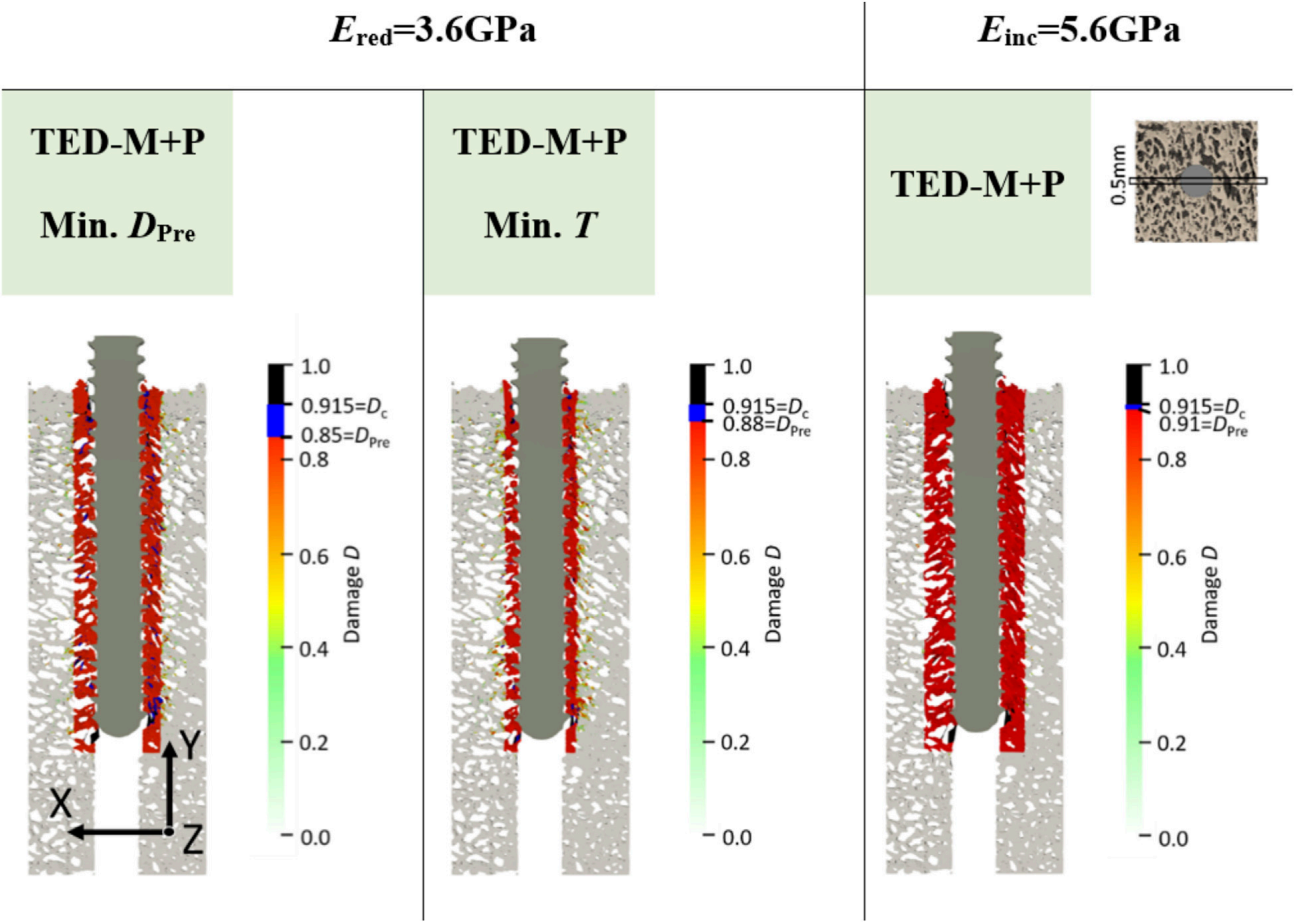
Damage at maximum force of one representative specimen (S10) for the two elastic moduli *E*_red_ = 3.6 GPa and *E*_inc_ = 5.6 GPa. All models included a simplified interface method (Stefanek et al., 2024; Steiner et al., 2017) with pre-damage (TED-M + P). A displacement scaling factor of 5 was used, and optimal pre-damage parameters were selected based on two criteria defined in Figure 3: minimal pre-damage (Min. *D*_Pre_) and minimal radial thickness criteria (Min. *T*). For the TED-M + P, the color bar includes blue regions between the pre-damage value *D*_Pre_ in the damage zone and the critical damage *D*_c_.

Overall, the models FB and TED-M exhibited similar damage distributions (see Figure 5). Most damage could be observed in bone material close to the screw but not directly attaching the screw core. The extent of damage reduced gradually from the screw axis towards the outer surface. However, a more detailed comparison revealed that the FB model displayed slightly higher damage and differences in the regions of critical damage. In contrast, the TED-M + P models all showed the implemented pre-damage zone close to the screw, with a radial thickness depending on the optimal pre-damage parameters (see Figures 5, 6). For TED-M + P models with *E*_red_ = 3.6GPa, screw pull-out led to a visible formation of additional damage around the predefined damage zone. In contrast, for TED-M + P models with *E* = 4.6 GPa and *E*_inc_ = 5.6GPa, the screw pull-out did not cause any additional damage outside the pre-damage zone.

## 4 Discussion

The goal of this study was to evaluate the agreement of screw pull-out force predictions of computationally efficient, materially nonlinear µFE models with experimental measurements, taking both contact interface and pre-damage into account. Across all µFE model variations - whether pre-damage or contact interface was implemented or not - the predicted maximum forces showed a strong correlation with experimental data. However, the selection of pre-damage parameters emerged as particularly critical for achieving quantitatively accurate predictions.

The optimal pre-damage parameters identified via evaluation of the mean heat map isolines generally aligned with previous studies. For an elastic modulus of 3.6 GPa and the investigated parameter range, the evaluated ideal radial thickness was between 0.3 mm and 0.63 mm, while for 4.6 GPa and 5.6 GPa, it was between 0.74 mm and 0.9 mm, which is similar to the radial thickness values selected from Steiner et al. (2017) (0.6 mm–0.9 mm) and Chevalier et al. (2018) (0.4 mm). Torcasio et al. (2012) and Steiner et al. (2017) selected higher damage values (>0.98) than the pre-damage values evaluated in this study (0.85–0.91). However, all compared studies differed from this study in various aspects (material law, contact implementations, predicted mechanical parameters). Furthermore, the pre-damage value in this study was restricted by the critical damage $D_{c}$ = 0.915 of the used material law. The heat maps revealed that radial thickness of the pre-damage zone, pre-damage value, and elastic modulus selection all influenced maximum force screw pull-out predictions. A higher radial thickness reduces the need for a high pre-damage value, and *vice versa*. In a similar manner, a lower modulus decreases the maximum force and allows for lower pre-damage parameters, while a higher modulus has the opposite effect. Hence, optimal pre-damage parameters are not unique and cannot be chosen independently. Accurate modeling of pre-damage requires identification of the correct, specimen-specific material properties as well as experimental determination of at least one pre-damage parameter (radial thickness or pre-damage value). In addition, it must be kept in mind that multiple other parameters of the µFE model (e.g., voxel size, contact model) and pre-processing steps (e.g., segmentation) can quantitatively affect maximum force predictions. Pre-damage parameters must therefore always be considered as specific for a given µFE modeling and workflow, rather than generally applicable.

All different interface and pre-damage combinations showed similar correlations (*R* ^2^ > 0.85) consistent with comparable studies by Ovesy et al. (2022) (*R* ^2^ > 0.91), Panagiotopoulou et al. (2021) (*R* ^2^ > 0.93) and Zhou et al. (2024) (*R* ^2^ = 0.79) using fully nonlinear models and standard commercial FE solvers. However, the models differed in their 1:1 fit to experimental maximum force results. The fully-bonded interface without pre-damage highly overestimated the maximum force predictions (MAPE = 61%), and the TED-M model only slightly improved the predictions (MAPE = 52%). In contrast, the TED-M + P models were able to improve the predictions and enabled a good 1:1 fit to experimental results, especially for *E*_red_ = 3.6 GPa and *E* = 4.6 GPa (MAPE<12%). The TED-M + P model for 5.6 GPa showed slightly higher errors (MAPE = 17%), likely due to a suboptimal elastic modulus selection. Although this study results suggest a strong impact of pre-damage modeling on screw pull-out force prediction, other studies still reached accurate results with good 1:1 correspondence without accounting for pre-damage at all (Ovesy et al., 2022; Panagiotopoulou et al., 2021; Zhou et al., 2024). Hence, simple nonlinear models without pre-damage implementations can still yield good correlations and 1:1 correspondence with experimental measurements on a structural level. This is in line with the results of this study, which showed that various modeling aspects (contact interaction, elastic modulus, pre-damage value and radial thickness) can be used to tune the 1:1 agreement of the models on a structural level (here: maximum pull-out force). However, especially for an in-depth analysis of bone-screw mechanical behavior beyond the structural level, these modeling aspects must be separated and correctly implemented.

Despite similar screw pull-out force predictions with various combinations of pre-damage parameters, differences between the models were evident in the damage distributions. FB and TED-M showed only slightly different damage distributions with damage gradually decreasing with larger distance from the screw. The pre-drilling reduced the influence of the contact interface on the damage distribution, as any potential contact at the screw tip was removed. Without pre-drilling, contact between the bone and the tip of the screw could cause large differences between fully bonded and contact models in a pull-out scenario (Stefanek et al., 2024). In contrast to FB and TED-M, TED-M + P models only predicted small amounts of damage outside the damage zone. This suggests that TED-M + P may overestimate damage within the damage zone while underestimating it outside. This error could be caused by the simple geometric representation of the cylindrical pre-damage zone with sharp boundaries, and should be further evaluated by comparison to experimentally measured pre-damage distributions. However, experimental methods for accurate measurement of pre-damage are not yet available and novel methods must be developed in order to validate µFE models at this level of detail. To guide researchers conducting similar studies, it is suggested to first perform a material parameter identification to establish these parameters as fixed. Subsequently, investigations into pre-damage formation caused by pre-drilling and screw insertion should be carried out. Using µCT scanning before and after screw insertion, the radial thickness of the pre-damage zone can be estimated (Steiner et al., 2016). The pre-damage value may be estimated using digital volume correlation, which provides detailed 3D displacement and strain measurements, helping to detect deformations beyond the yield limit (Hussein et al., 2012; Peña Fernández et al., 2020; 2021; Xu, 2018). Additionally, staining techniques can highlight crack formation, enabling to assess the extent of damage (Burr et al., 1998; Lee et al., 2003). Furthermore, extensive calibration studies involving multiple loading cases, specimens, and screws could be conducted to back-calculate the pre-damage parameters (Steiner et al., 2017).

The study has several limitations. To begin with, the elastic modulus was taken from literature and was not experimentally determined for the used specimens. Furthermore, the applied nonlinear material properties were based on human bones and only adapted to porcine bones (Stipsitz et al., 2020). As the pre-damage parameters as well as the maximum force predictions depend on the selected material properties, this study cannot provide optimal parameters for future studies, but can only show the effect and ambiguity of the pre-damage parameters. Additionally, the study outcomes were restricted to pre-drilled bone samples. The drilling process leads to reduced strength and stability of the bone-screw construct (Pandey and Panda, 2013) and leaves bone debris in the pilot-holes, which might be interpreted as intact bone in the µFE models. Furthermore, all results were limited to a single loading case, a single screw with one insertion depth, and the nonlinear material model of Stipsitz et al. (2020), developed for efficient µFE simulations.

In conclusion, screw pull-out forces predicted by computationally efficient, materially nonlinear µFE are highly correlated with experimental measurements, even with a fully bonded interface and without considering pre-damage. However, to obtain quantitatively accurate results, careful orchestration of contact modeling, material properties and pre-damage parameters is required. The selection of these parameters is ambiguous and experimental assessment of pre-damage distributions is necessary to further refine and validate the µFE models. µCT scanning before and after screw insertion could be valuable in providing more accurate pre-damage distributions while methods like digital volume correlation and staining may help to estimate the pre-damage value. Until such experimental data become available, optimal parameters of a simplified pre-damage model must be identified as proposed in this study using comparison to experimental results.

## Data Availability

The raw data supporting the conclusions of this article will be made available by the authors, without undue reservation.

## Abbreviations

FB, fully bonded interface model without pre-damage; TED-M, simplified contact interface model without pre-damage; TED-M + P, simplified contact interface model with pre-damage; *T*, radial thickness of damage zone; *D*_*Pre*_, damage value inside damage zone; Min. *T*, minimal radial thickness criteria; Min. *D*_*Pre*_, minimal damage value criteria.
