# Supplementary material for: Screw pull-out force predictions in porcine radii using efficient nonlinear µFE models including contact and pre-damage
Source: Front Bioeng Biotechnol. 2025 Mar 24;13:1524235. doi: 10.3389/fbioe.2025.1524235 (PMC11973284; doi:10.3389/fbioe.2025.1524235)
Supplement: Supplementary file 1 [file DataSheet1.docx]

**Supplementary material**

**S1. Force vs. Displacement and heat maps**

|  | **S1** | | | |
| --- | --- | --- | --- | --- |
| 1. **Experiment: Force - Displacement** | 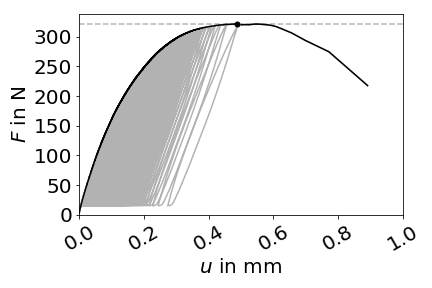 | | ***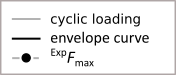*** | |
|  | ***E*_red_=3.6GPa** | ***E*=4.6GPa** | | ***E*_inc_=5.6GPa** |
| **(B) Simulation: Force - Displacement** | 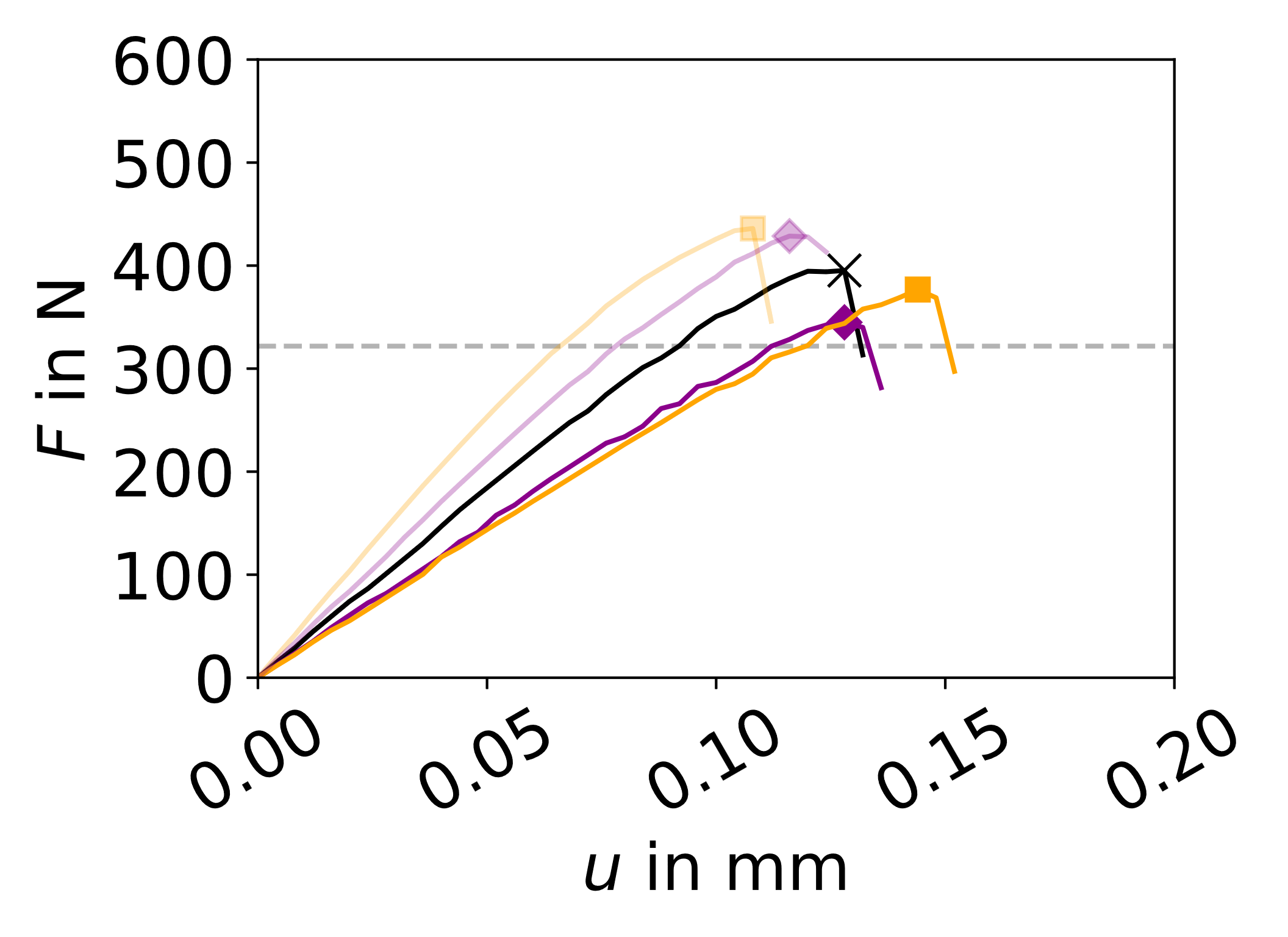 | 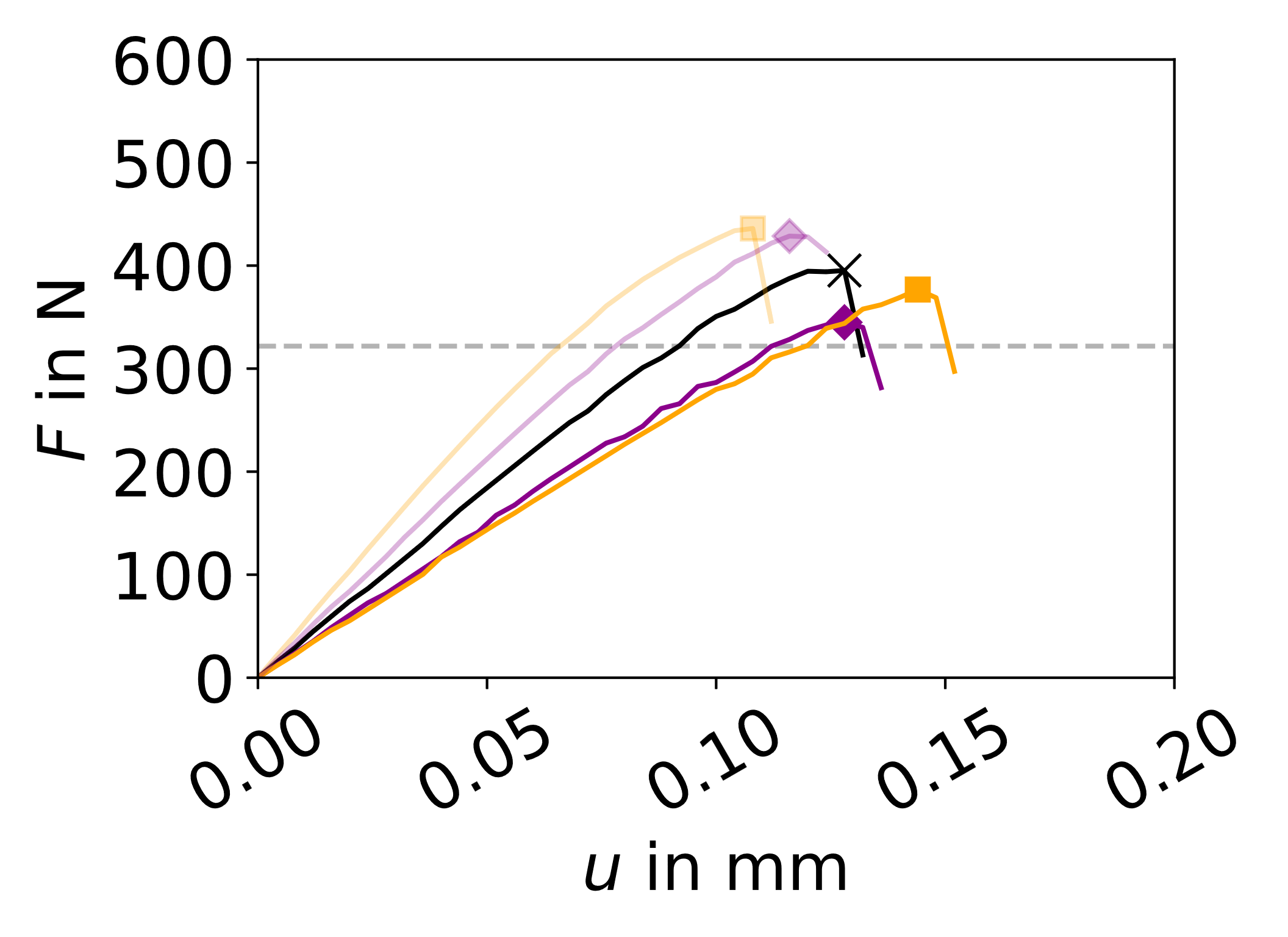 | | 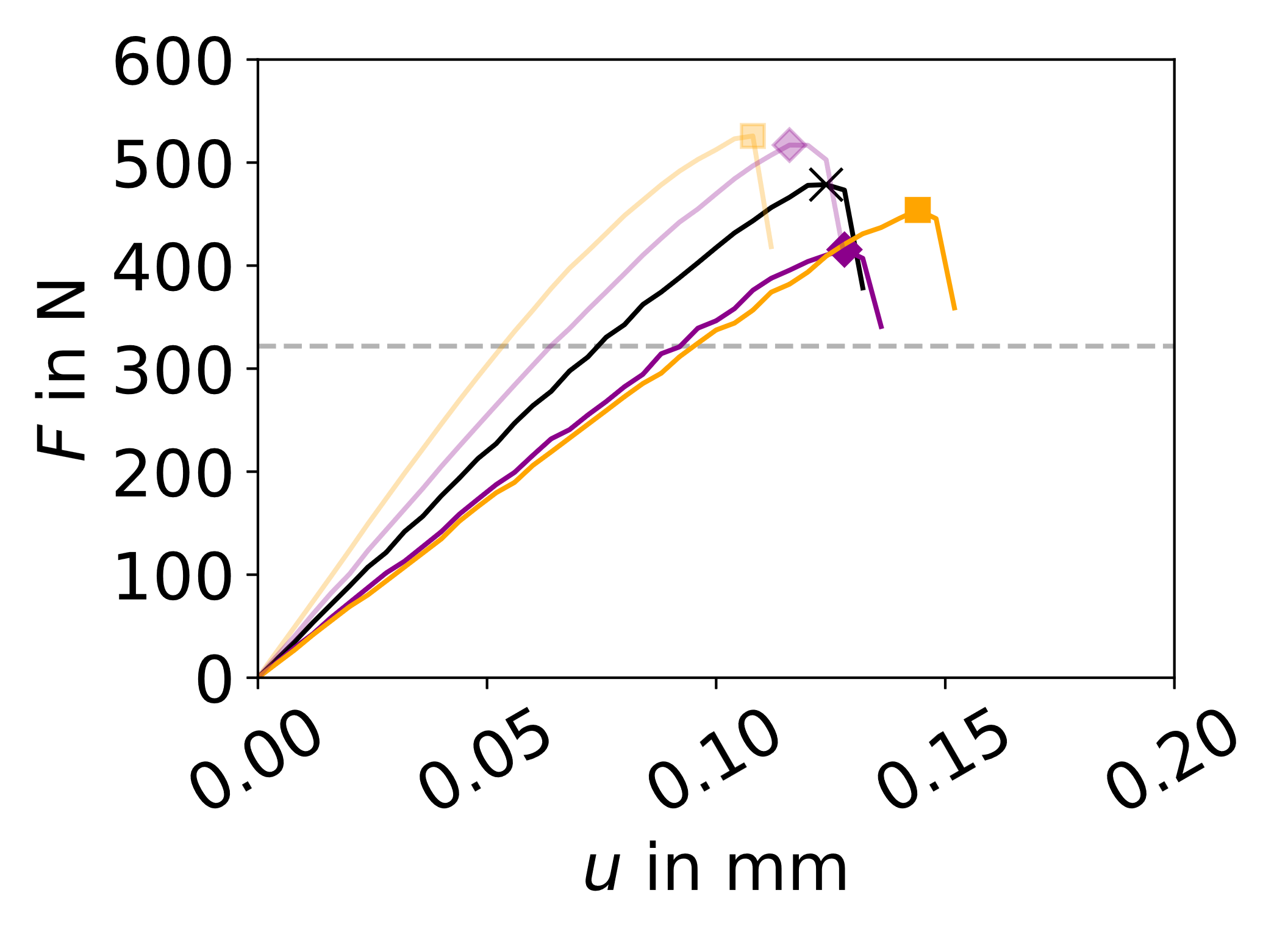 |
|  | ***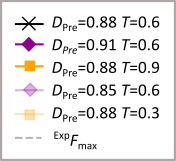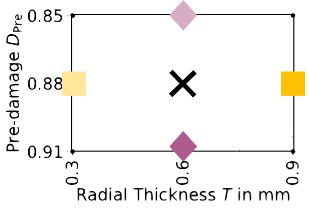*** | | | |
| **(C) Heat Maps** | 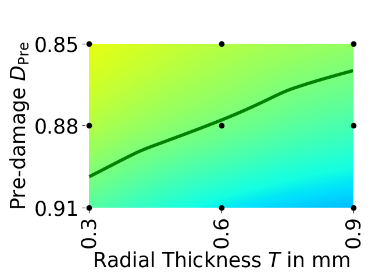 | 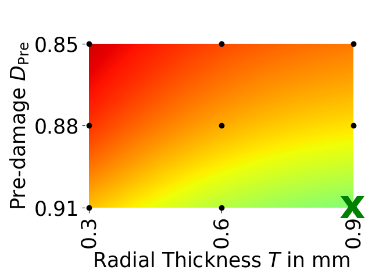 | | 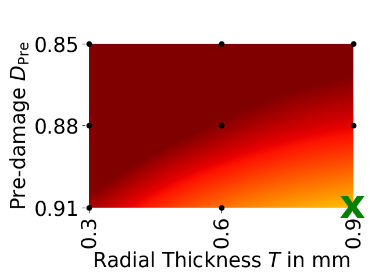 |
|  | 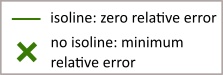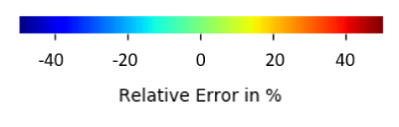 | | | |
| **Fig. S1.1:** Experimental force-displacement curve (**A**), simulated force displacement curves for different values of radial thickness *T* and pre-damage value *D*_Pre_ (**B**) and heat maps showing the relative error in maximum force (**C**) of specimen S1. Simulated force-displacement curves and heat maps are shown for three different elastic moduli of bone material *E*_red_=3.6GPa, *E*=4.6GPa, and *E*_inc_=5.6GPa. The simulated force-displacement curves (**B**) show a selection of five parameter combinations of *T* and *D*_Pre_. In the heat maps (**C**), green isolines mark the parameter combinations of pre-damage *D*_Pre_ and radial thickness of damage zone *T*, where the relative error in maximum force between simulation and experiment is zero. In case that no parameter combination can be found that leads to zero relative error, the parameter combination where the relative error is minimal is marked by a green cross. | | | | |

|  | **S2** | | | |
| --- | --- | --- | --- | --- |
| 1. **Experiment: Force - Displacement** | 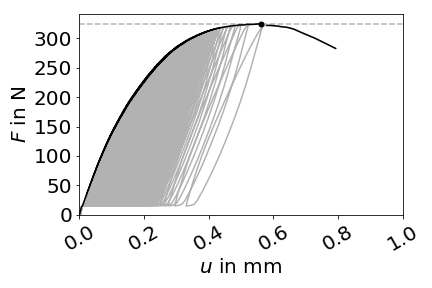 | | ***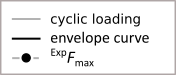*** | |
|  | ***E*_red_=3.6GPa** | ***E*=4.6GPa** | | ***E*_inc_=5.6GPa** |
| **(B) Simulation: Force - Displacement** | 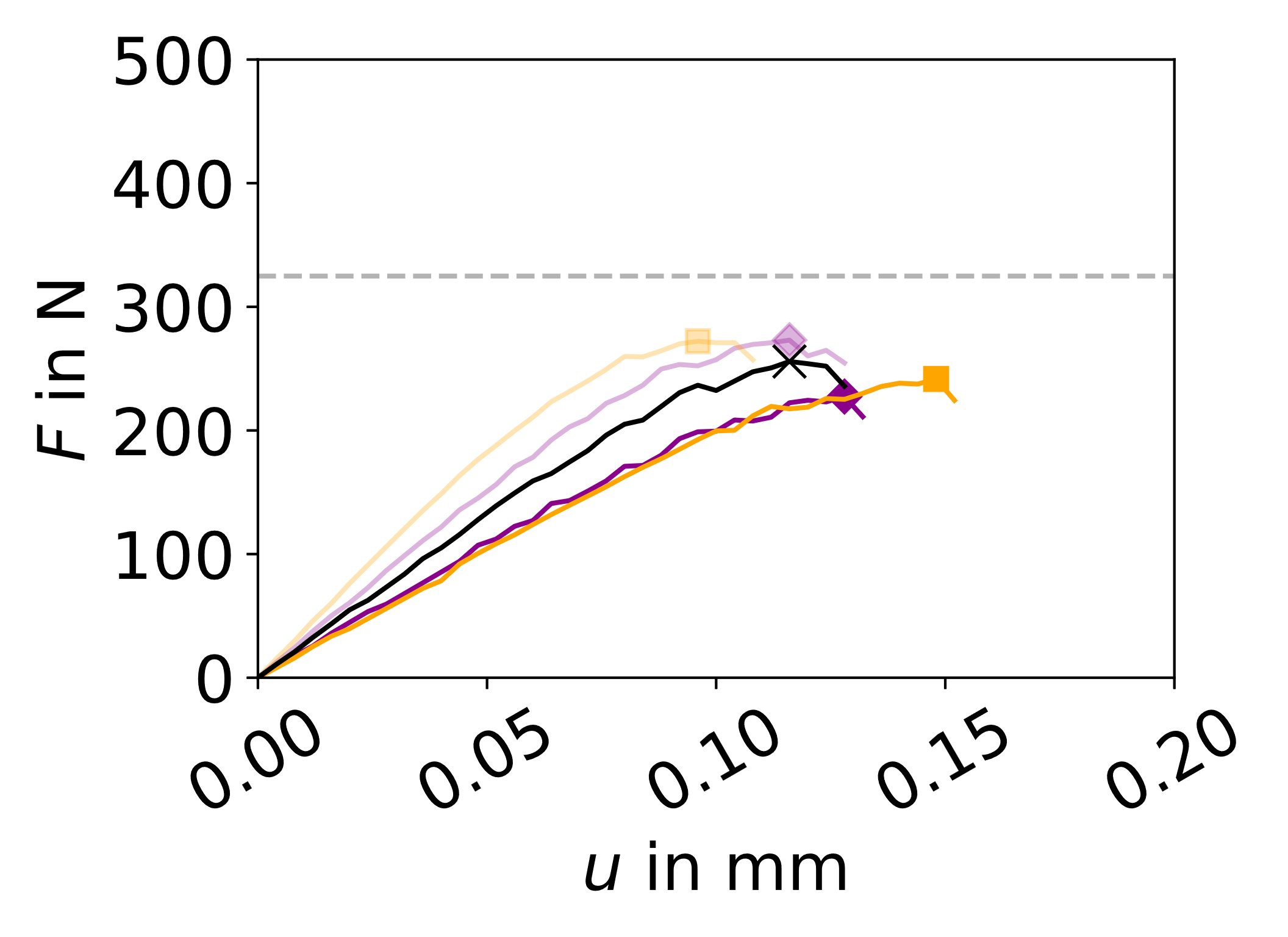 | 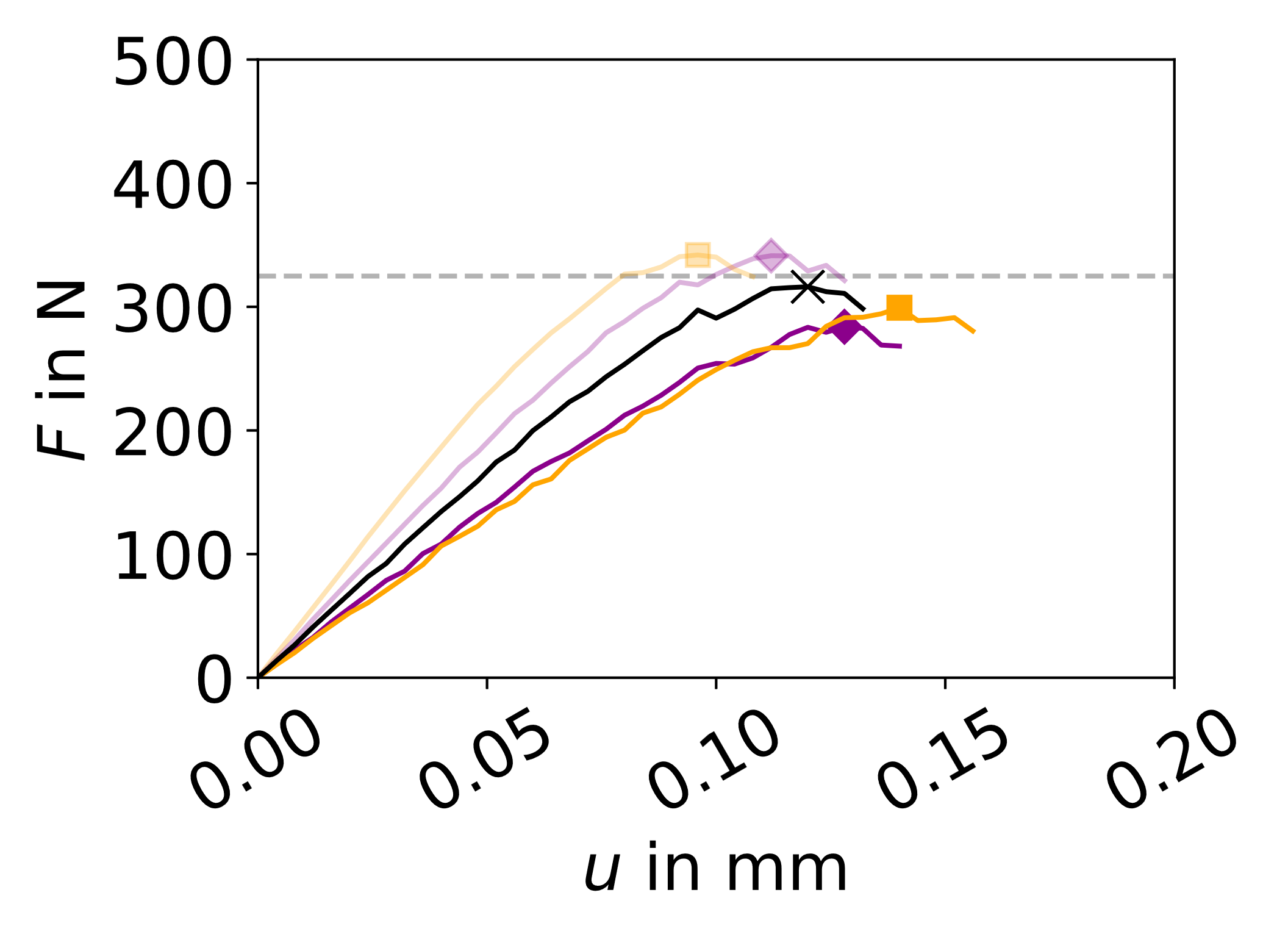 | | 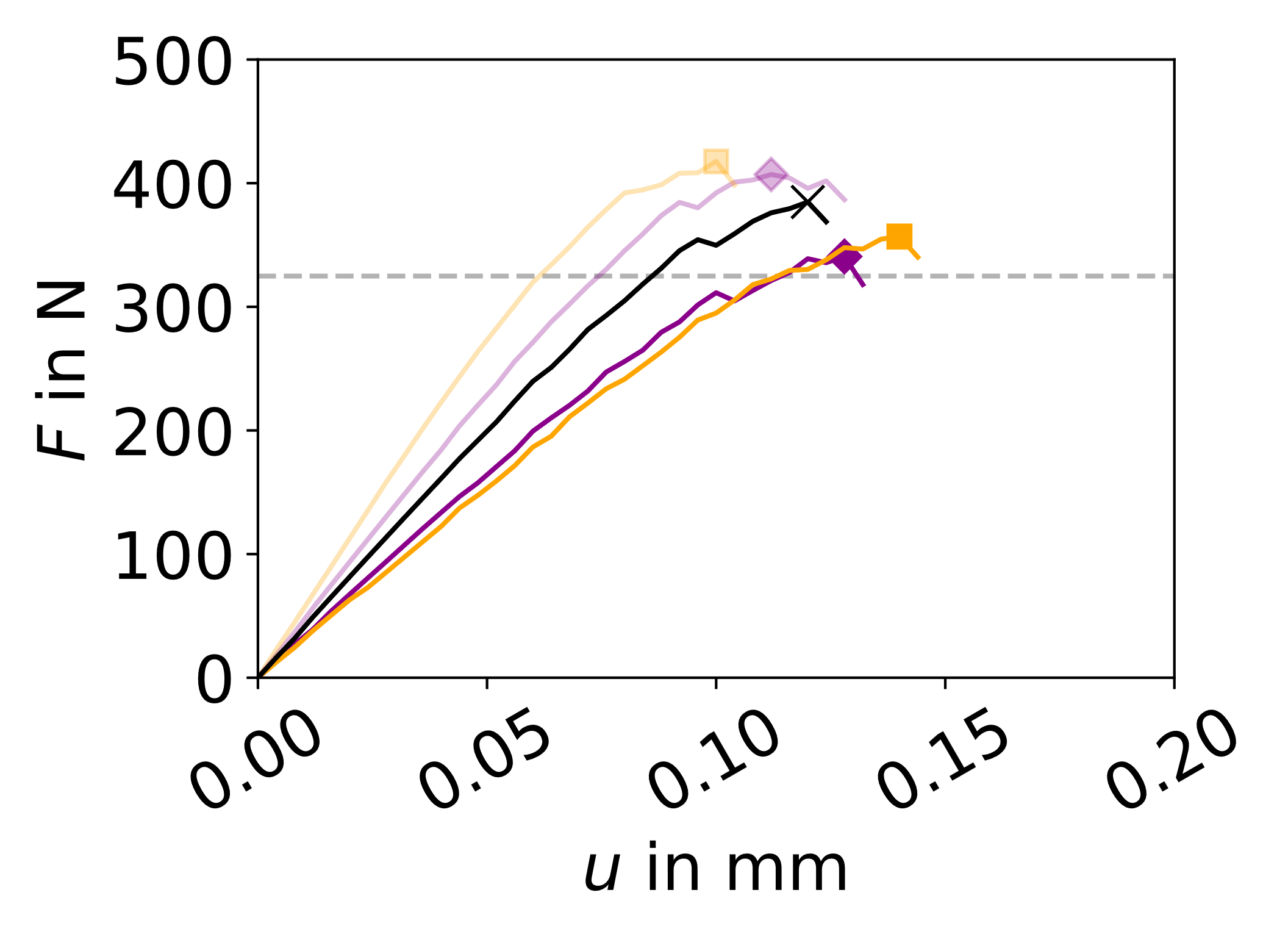 |
|  | ***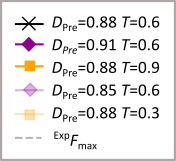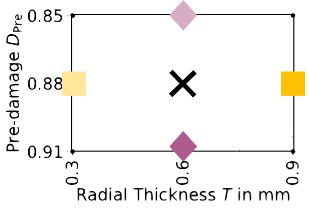*** | | | |
| **(C) Heat Maps** | 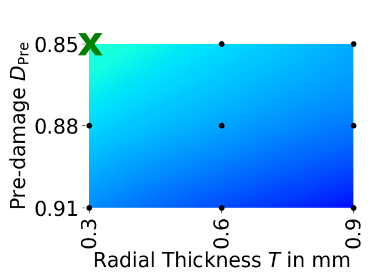 | 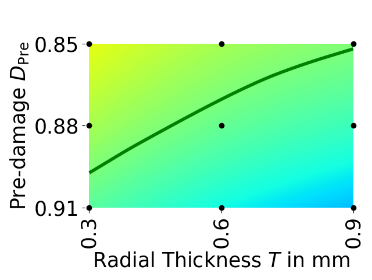 | | 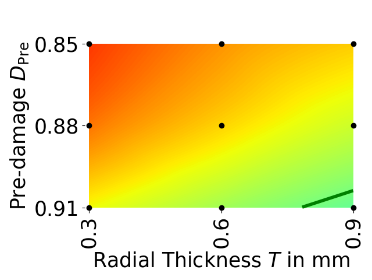 |
|  | 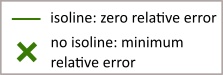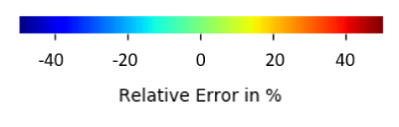 | | | |
| **Fig. S1.2:** Experimental force-displacement curve (**A**), simulated force displacement curves for different values of radial thickness *T* and pre-damage value *D*_Pre_ (**B**) and heat maps showing the relative error in maximum force (**C**) of specimen S2. Simulated force-displacement curves and heat maps are shown for three different elastic moduli of bone material *E*_red_=3.6GPa, *E*=4.6GPa, and *E*_inc_=5.6GPa. The simulated force-displacement curves (**B**) show a selection of five parameter combinations of *T* and *D*_Pre_. In the heat maps (**C**), green isolines mark the parameter combinations of pre-damage *D*_Pre_ and radial thickness of damage zone *T*, where the relative error in maximum force between simulation and experiment is zero. In case that no parameter combination can be found that leads to zero relative error, the parameter combination where the relative error is minimal is marked by a green cross. | | | | |

|  | **S3** | | | |
| --- | --- | --- | --- | --- |
| 1. **Experiment: Force - Displacement** | 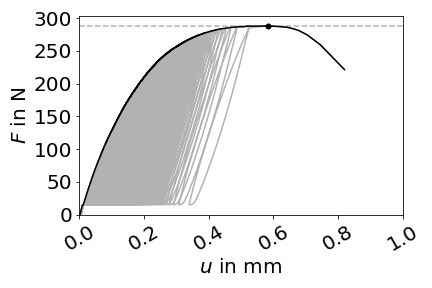 | | ***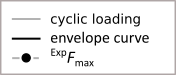*** | |
|  | ***E*_red_=3.6GPa** | ***E*=4.6GPa** | | ***E*_inc_=5.6GPa** |
| **(B) Simulation: Force - Displacement** | 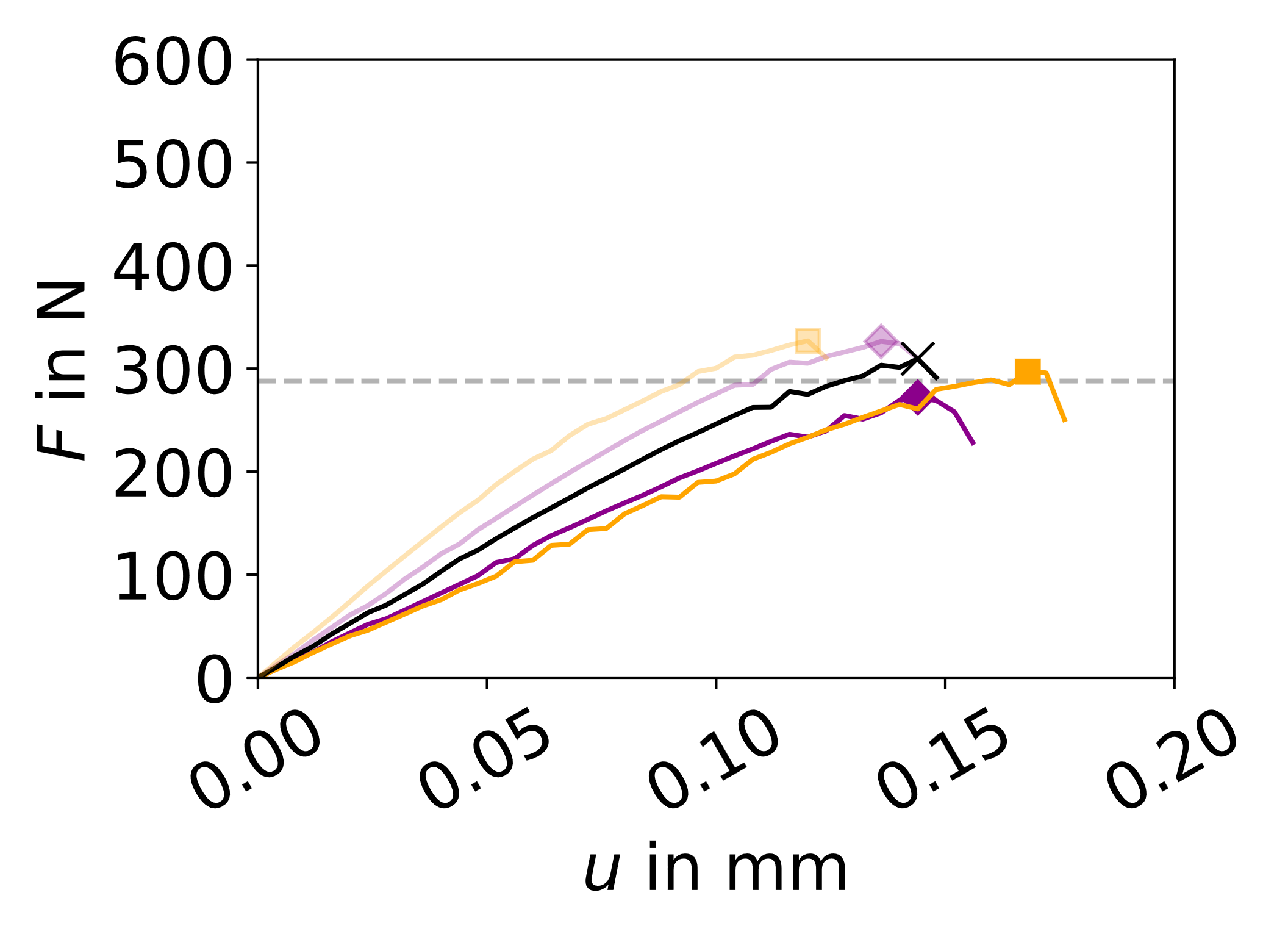 | 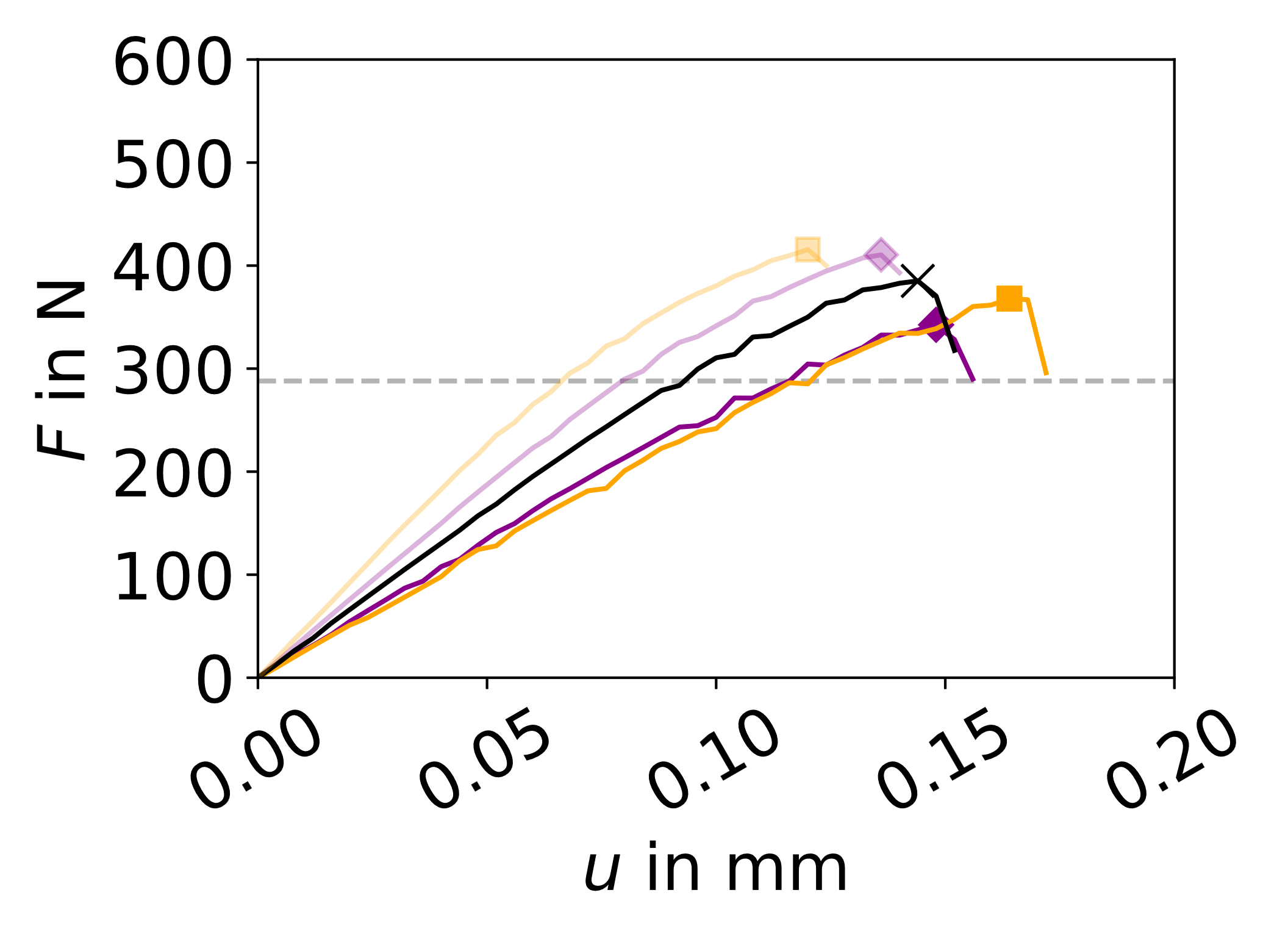 | | v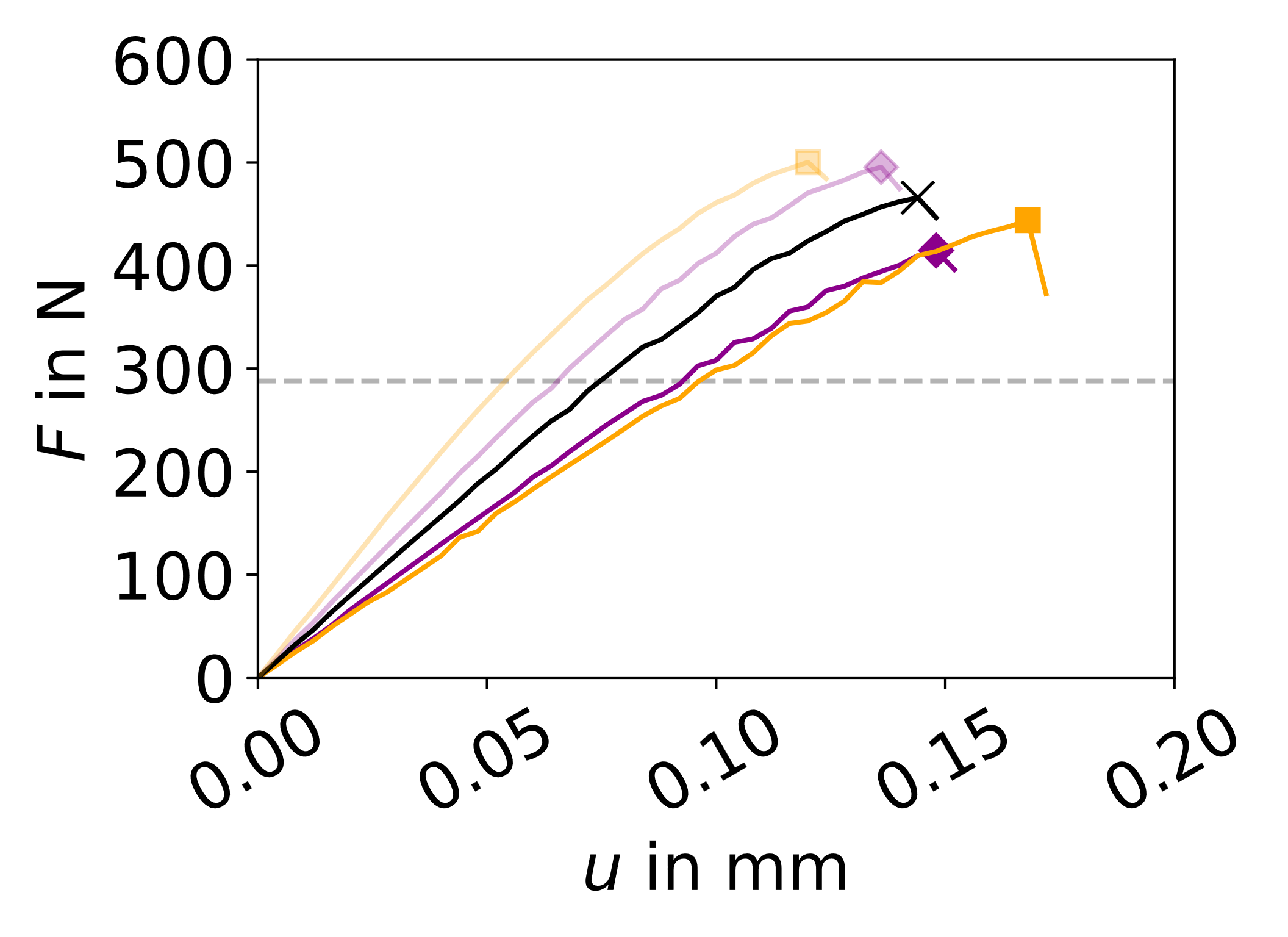 |
|  | ***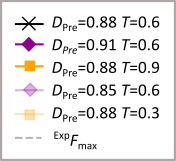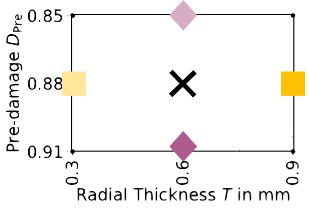*** | | | |
| **(C) Heat Maps** | 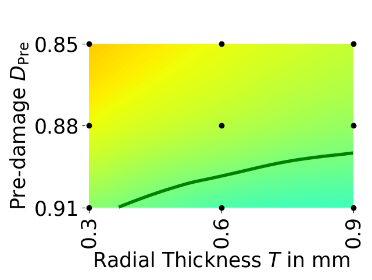 | 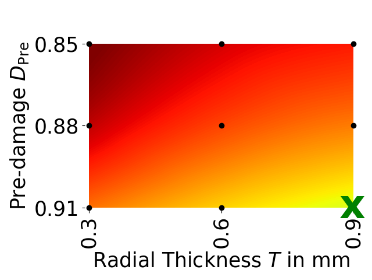 | | 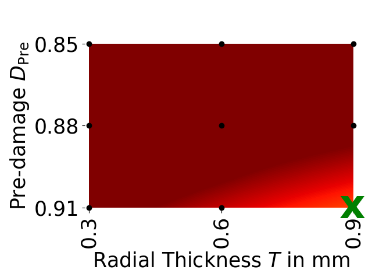 |
|  | 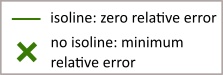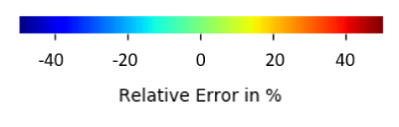 | | | |
| **Fig. S1.3:** Experimental force-displacement curve (**A**), simulated force displacement curves for different values of radial thickness *T* and pre-damage value *D*_Pre_ (**B**) and heat maps showing the relative error in maximum force (**C**) of specimen S3. Simulated force-displacement curves and heat maps are shown for three different elastic moduli of bone material *E*_red_=3.6GPa, *E*=4.6GPa, and *E*_inc_=5.6GPa. The simulated force-displacement curves (**B**) show a selection of five parameter combinations of *T* and *D*_Pre_. In the heat maps (**C**), green isolines mark the parameter combinations of pre-damage *D*_Pre_ and radial thickness of damage zone *T*, where the relative error in maximum force between simulation and experiment is zero. In case that no parameter combination can be found that leads to zero relative error, the parameter combination where the relative error is minimal is marked by a green cross. | | | | |

|  | **S4** | | | |
| --- | --- | --- | --- | --- |
| 1. **Experiment: Force - Displacement** | 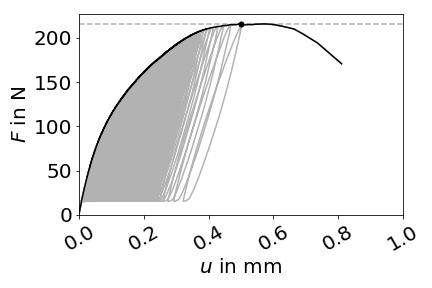 | | ***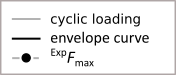*** | |
|  | ***E*_red_=3.6GPa** | ***E*=4.6GPa** | | ***E*_inc_=5.6GPa** |
| **(B) Simulation: Force - Displacement** | 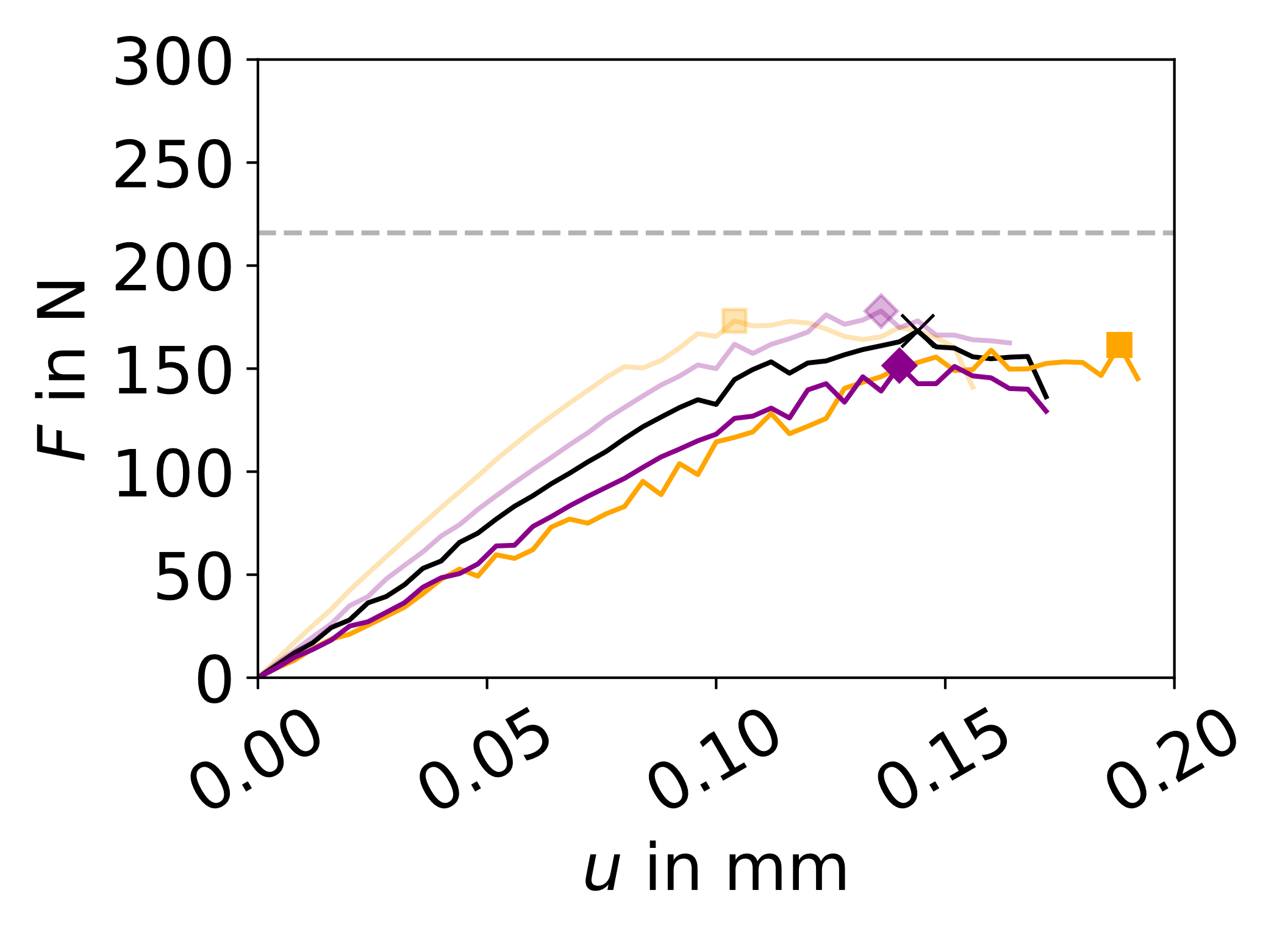 | 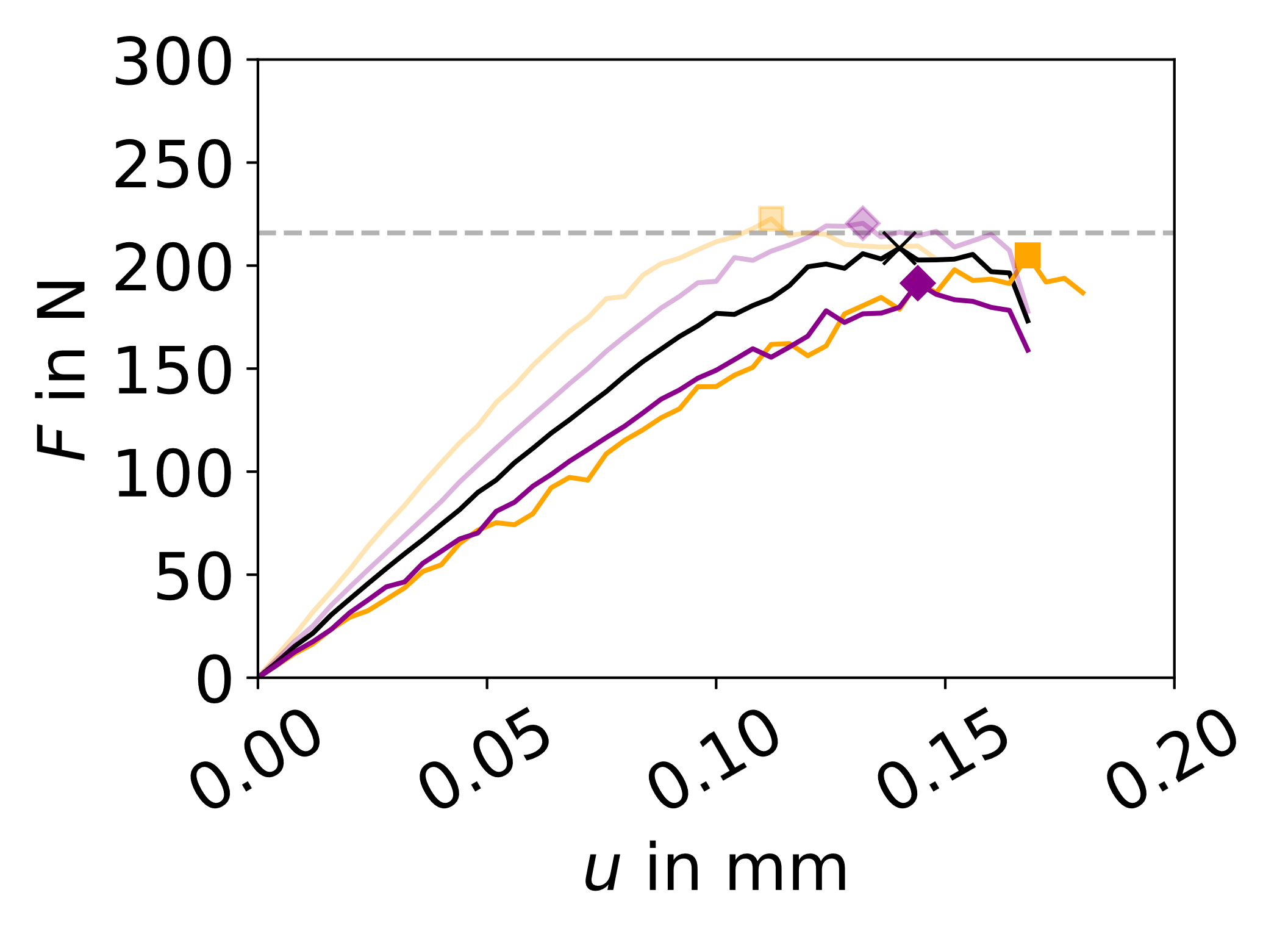 | | 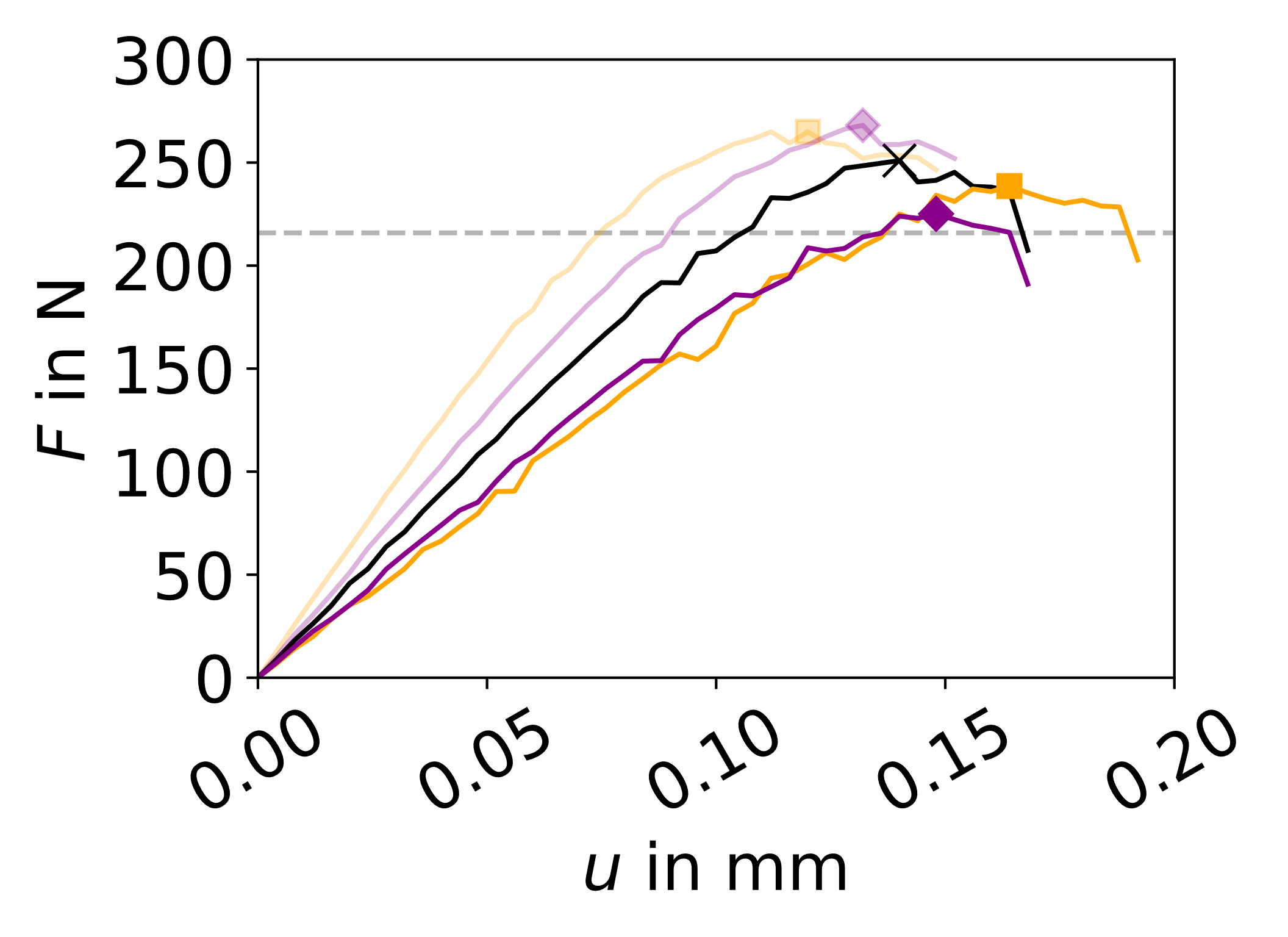 |
|  | ***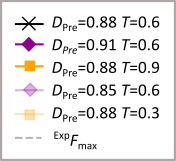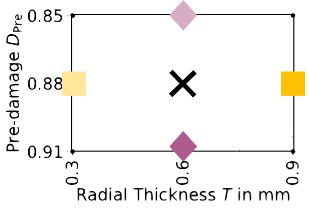*** | | | |
| **(C) Heat Maps** | 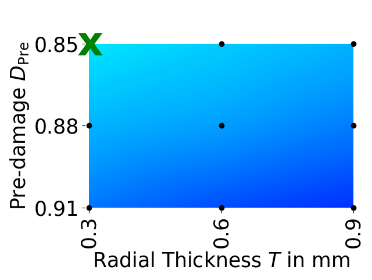 | 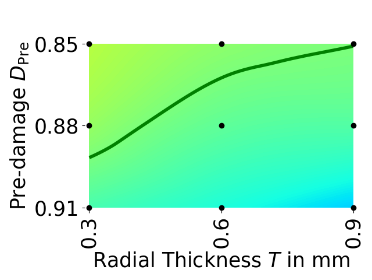 | | 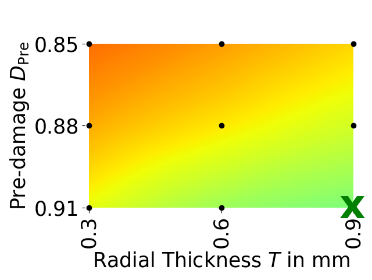v |
|  | 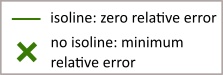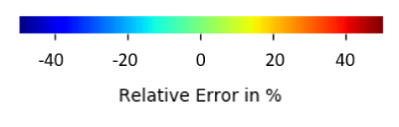 | | | |
| **Fig. S1.4:** Experimental force-displacement curve (**A**), simulated force displacement curves for different values of radial thickness *T* and pre-damage value *D*_Pre_ (**B**) and heat maps showing the relative error in maximum force (**C**) of specimen S4. Simulated force-displacement curves and heat maps are shown for three different elastic moduli of bone material *E*_red_=3.6GPa, *E*=4.6GPa, and *E*_inc_=5.6GPa. The simulated force-displacement curves (**B**) show a selection of five parameter combinations of *T* and *D*_Pre_. In the heat maps (**C**), green isolines mark the parameter combinations of pre-damage *D*_Pre_ and radial thickness of damage zone *T*, where the relative error in maximum force between simulation and experiment is zero. In case that no parameter combination can be found that leads to zero relative error, the parameter combination where the relative error is minimal is marked by a green cross. | | | | |

|  | **S5** | | | |
| --- | --- | --- | --- | --- |
| 1. **Experiment: Force - Displacement** | 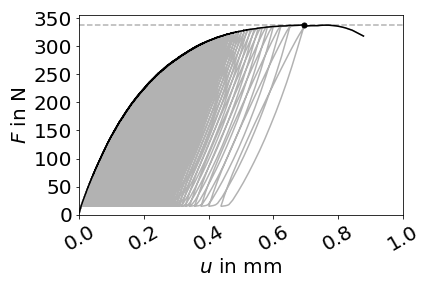 | | ***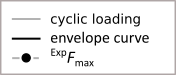*** | |
|  | ***E*_red_=3.6GPa** | ***E*=4.6GPa** | | ***E*_inc_=5.6GPa** |
| **(B) Simulation: Force - Displacement** | 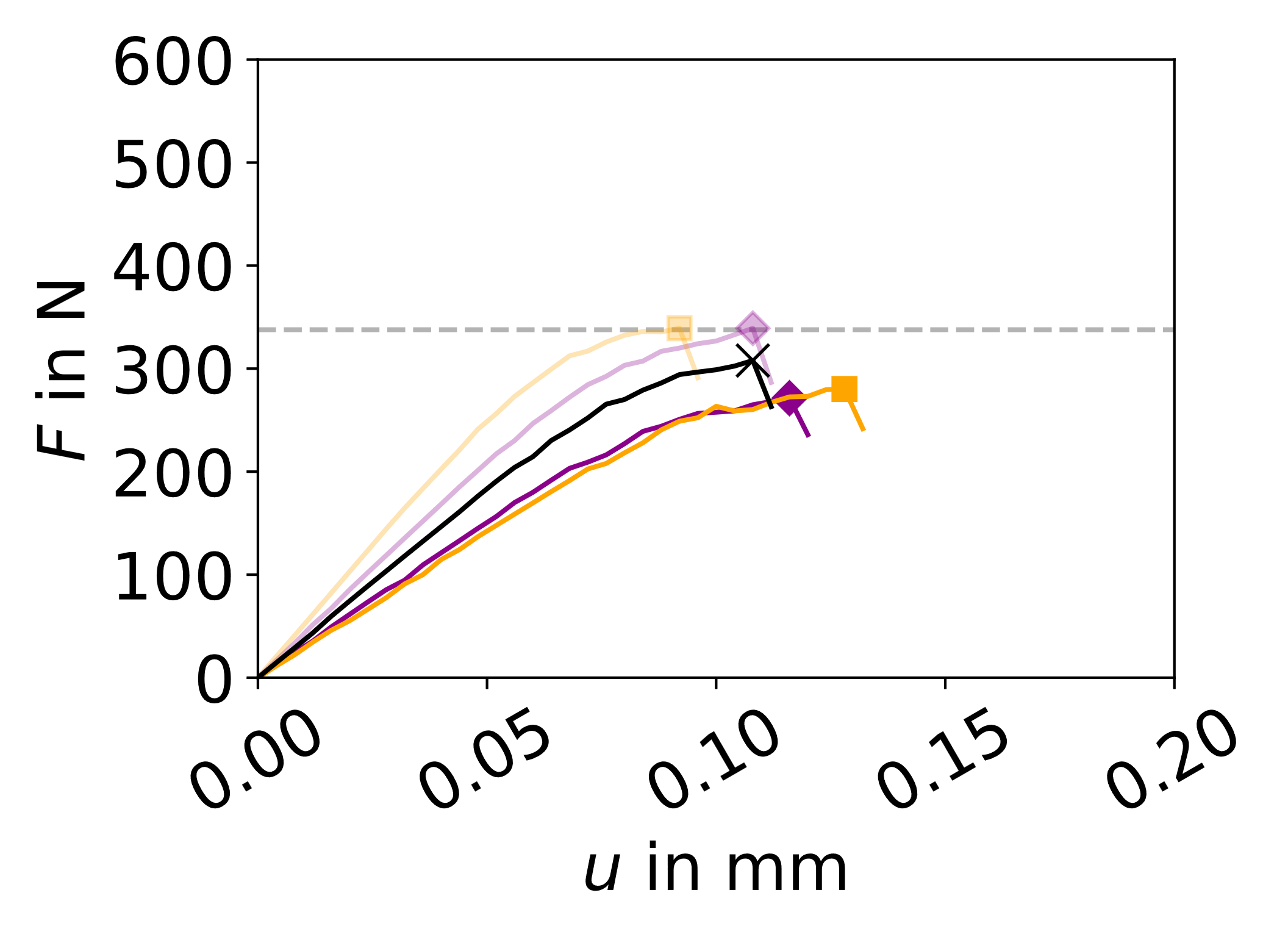 | 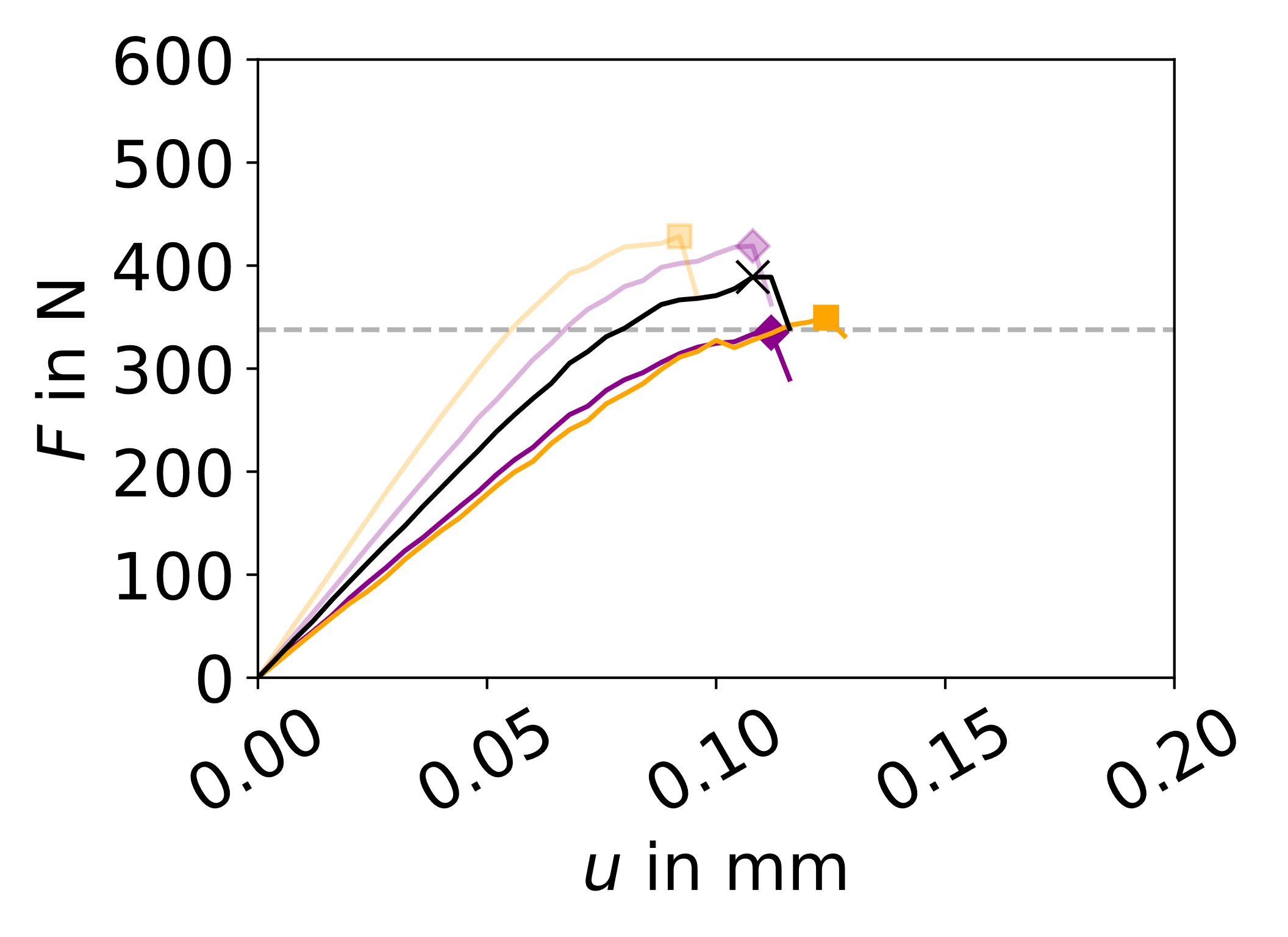 | | 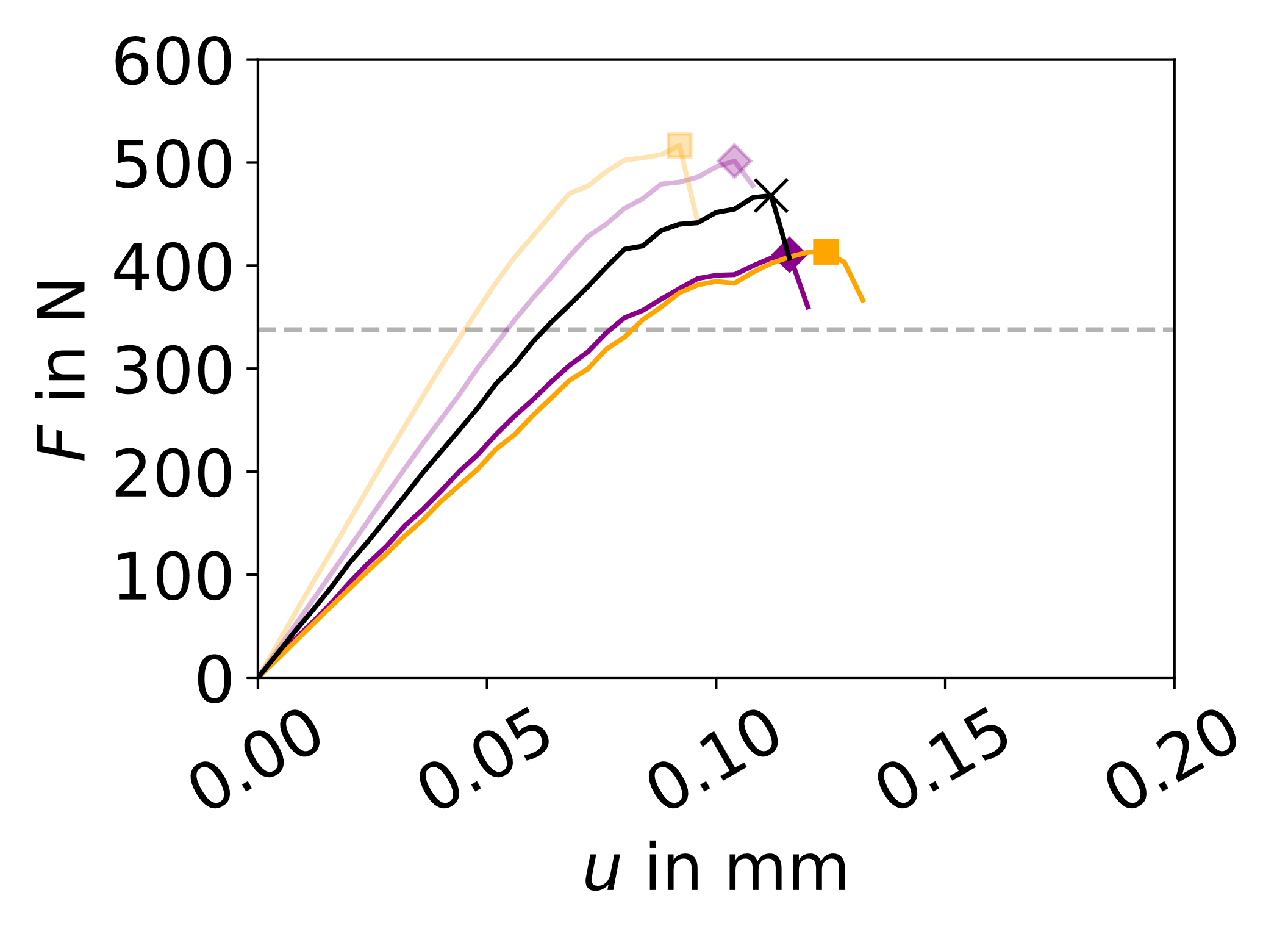 |
|  | ***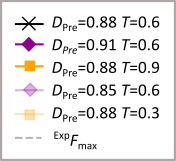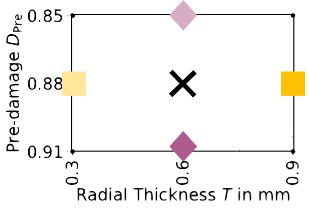*** | | | |
| **(C) Heat Maps** | 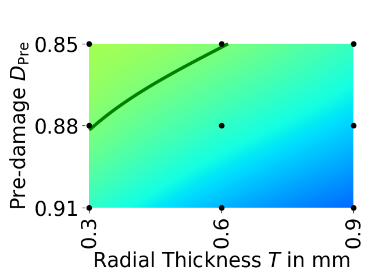 | 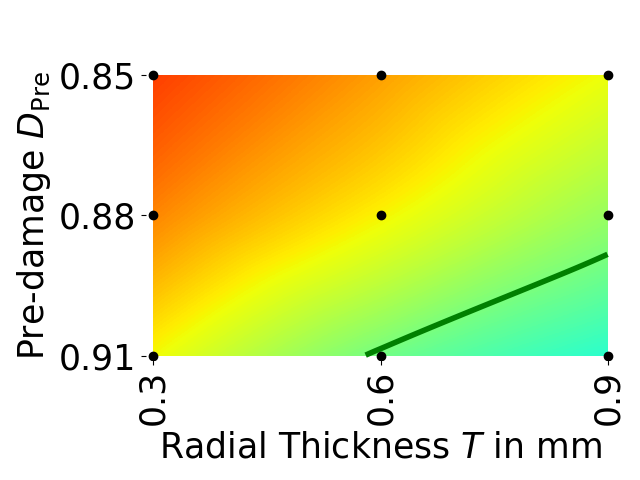 | | 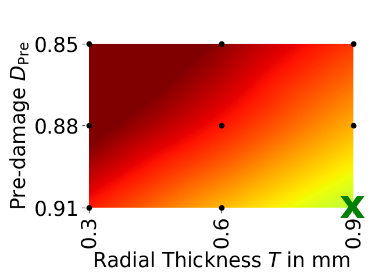 |
|  | 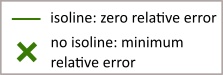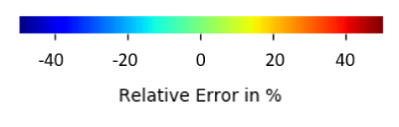 | | | |
| **Fig. S1.5:** Experimental force-displacement curve (**A**), simulated force displacement curves for different values of radial thickness *T* and pre-damage value *D*_Pre_ (**B**) and heat maps showing the relative error in maximum force (**C**) of specimen S5. Simulated force-displacement curves and heat maps are shown for three different elastic moduli of bone material *E*_red_=3.6GPa, *E*=4.6GPa, and *E*_inc_=5.6GPa. The simulated force-displacement curves (**B**) show a selection of five parameter combinations of *T* and *D*_Pre_. In the heat maps (**C**), green isolines mark the parameter combinations of pre-damage *D*_Pre_ and radial thickness of damage zone *T*, where the relative error in maximum force between simulation and experiment is zero. In case that no parameter combination can be found that leads to zero relative error, the parameter combination where the relative error is minimal is marked by a green cross. | | | | |

|  | **S6** | | | |
| --- | --- | --- | --- | --- |
| 1. **Experiment: Force - Displacement** | 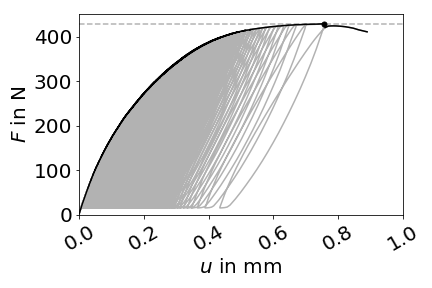 | | ***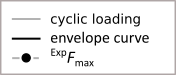*** | |
|  | ***E*_red_=3.6GPa** | ***E*=4.6GPa** | | ***E*_inc_=5.6GPa** |
| **(B) Simulation: Force - Displacement** | 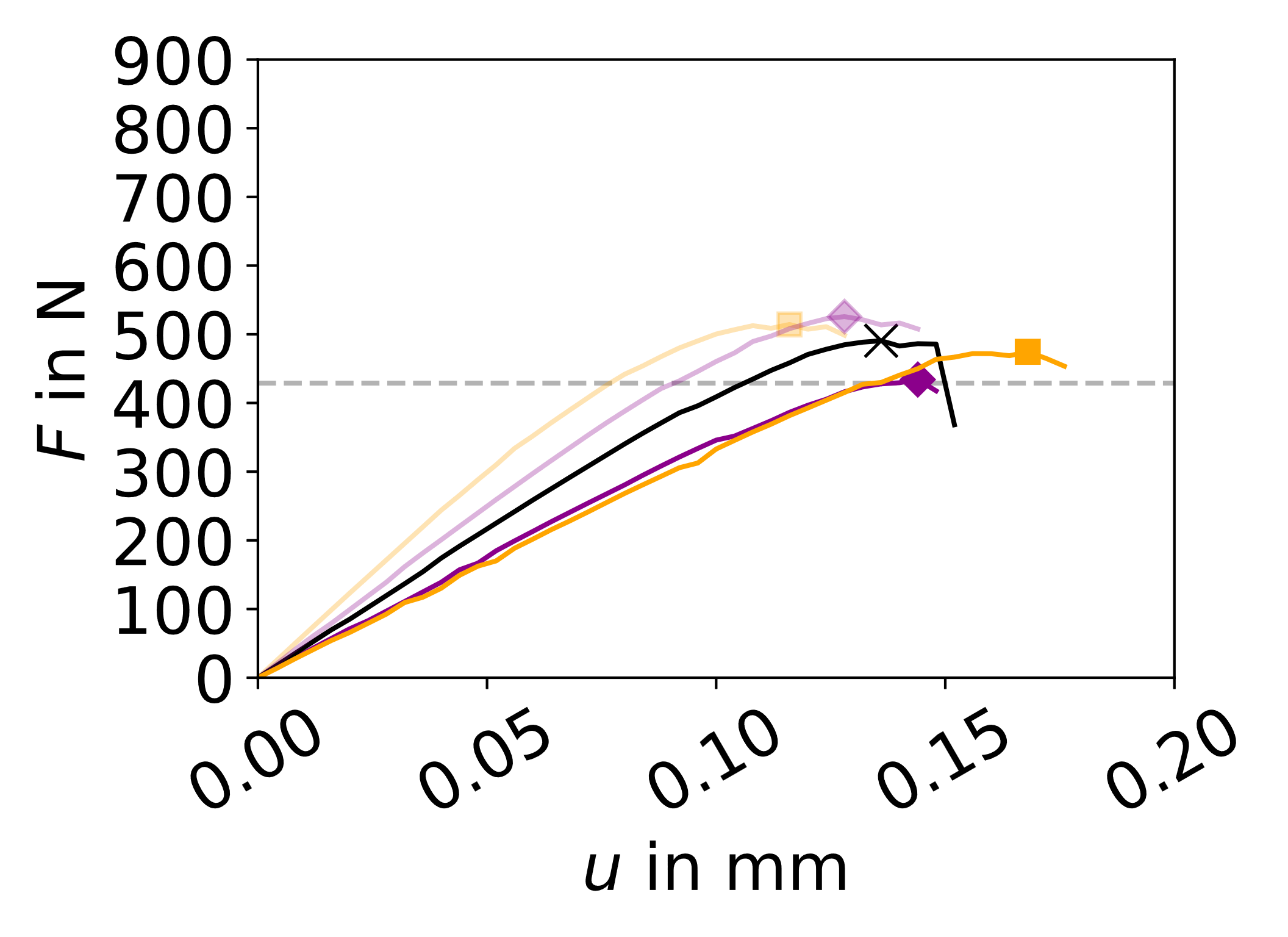 | 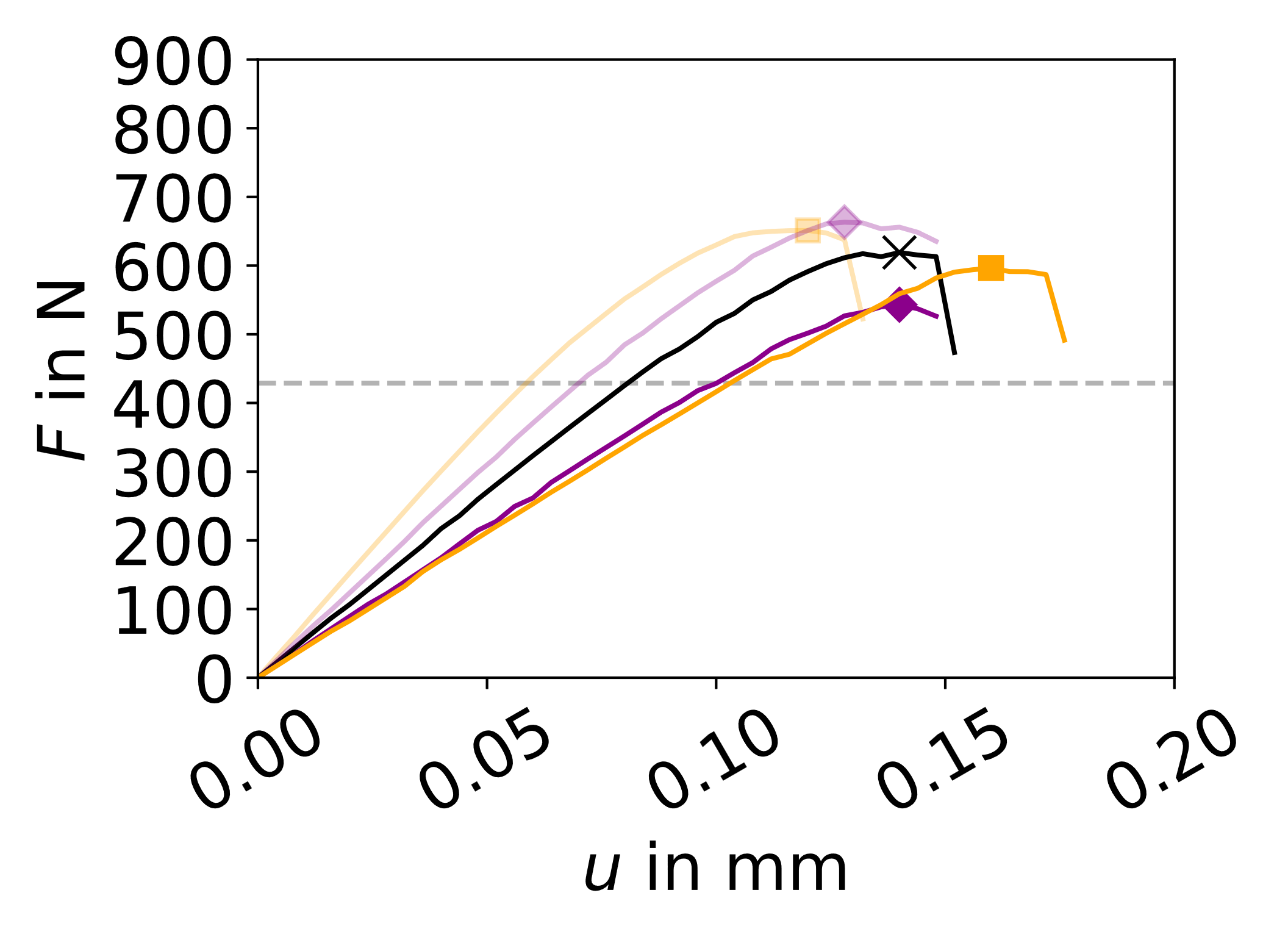 | | 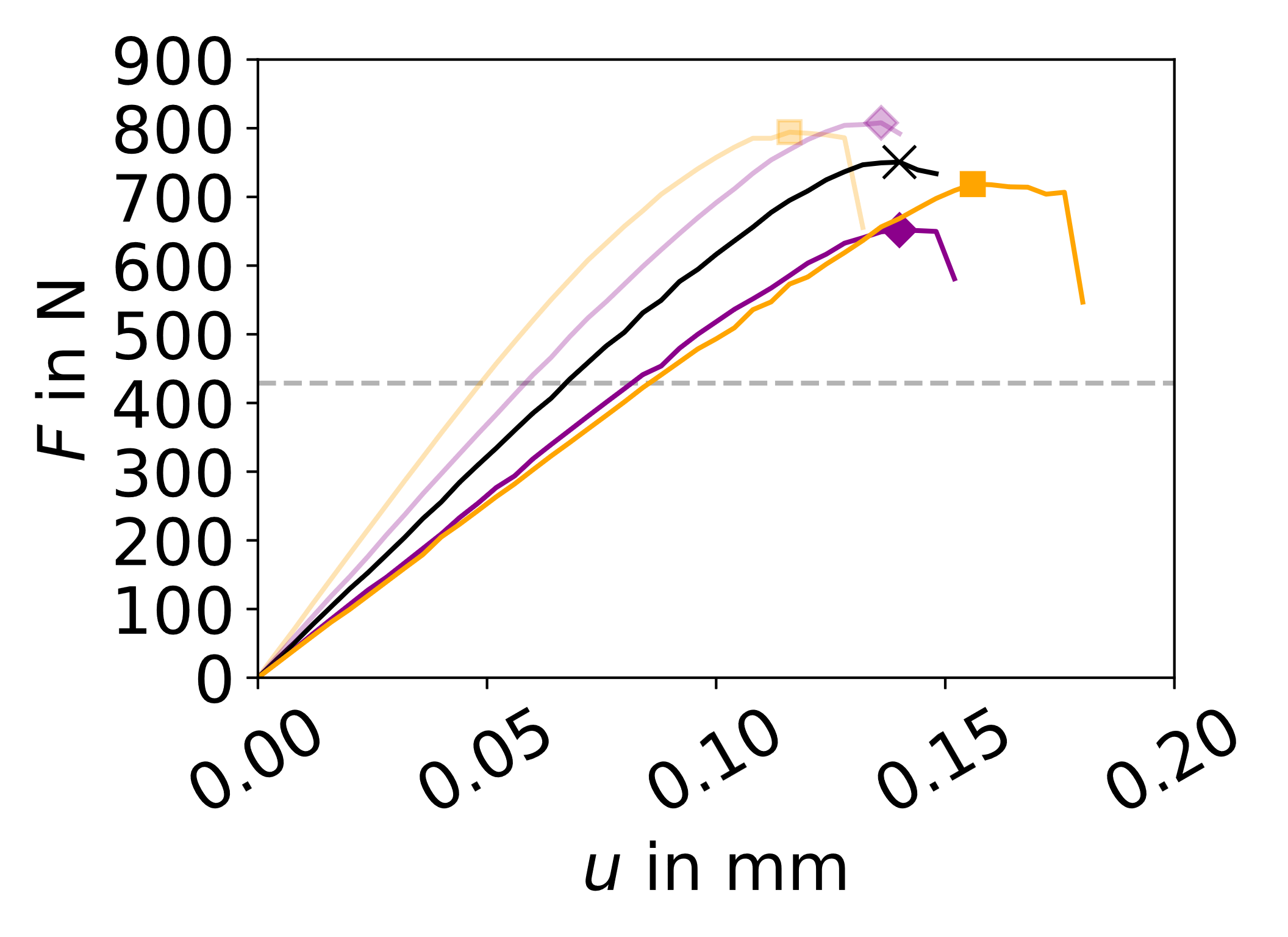 |
|  | ***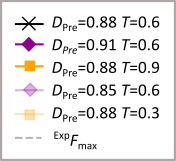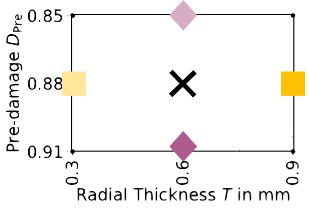*** | | | |
| **(C) Heat Maps** | 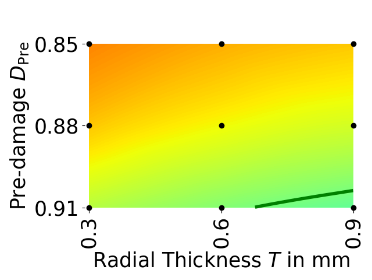 | 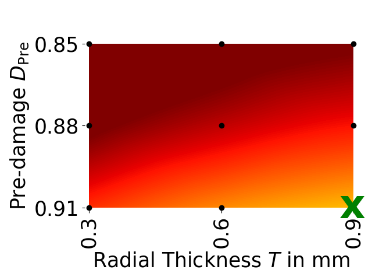 | | 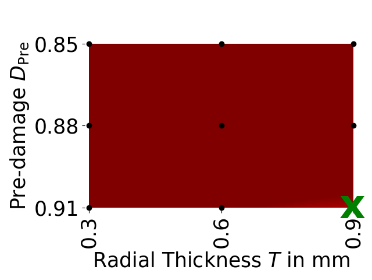 |
|  | 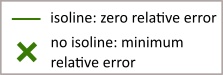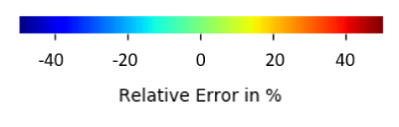 | | | |
| **Fig. S1.6:** Experimental force-displacement curve (**A**), simulated force displacement curves for different values of radial thickness *T* and pre-damage value *D*_Pre_ (**B**) and heat maps showing the relative error in maximum force (**C**) of specimen S6. Simulated force-displacement curves and heat maps are shown for three different elastic moduli of bone material *E*_red_=3.6GPa, *E*=4.6GPa, and *E*_inc_=5.6GPa. The simulated force-displacement curves (**B**) show a selection of five parameter combinations of *T* and *D*_Pre_. In the heat maps (**C**), green isolines mark the parameter combinations of pre-damage *D*_Pre_ and radial thickness of damage zone *T*, where the relative error in maximum force between simulation and experiment is zero. In case that no parameter combination can be found that leads to zero relative error, the parameter combination where the relative error is minimal is marked by a green cross. | | | | |

|  | **S7** | | | |
| --- | --- | --- | --- | --- |
| 1. **Experiment: Force - Displacement** | 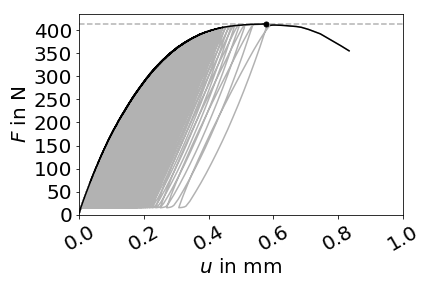 | | ***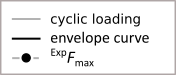*** | |
|  | ***E*_red_=3.6GPa** | ***E*=4.6GPa** | | ***E*_inc_=5.6GPa** |
| **(B) Simulation: Force - Displacement** | 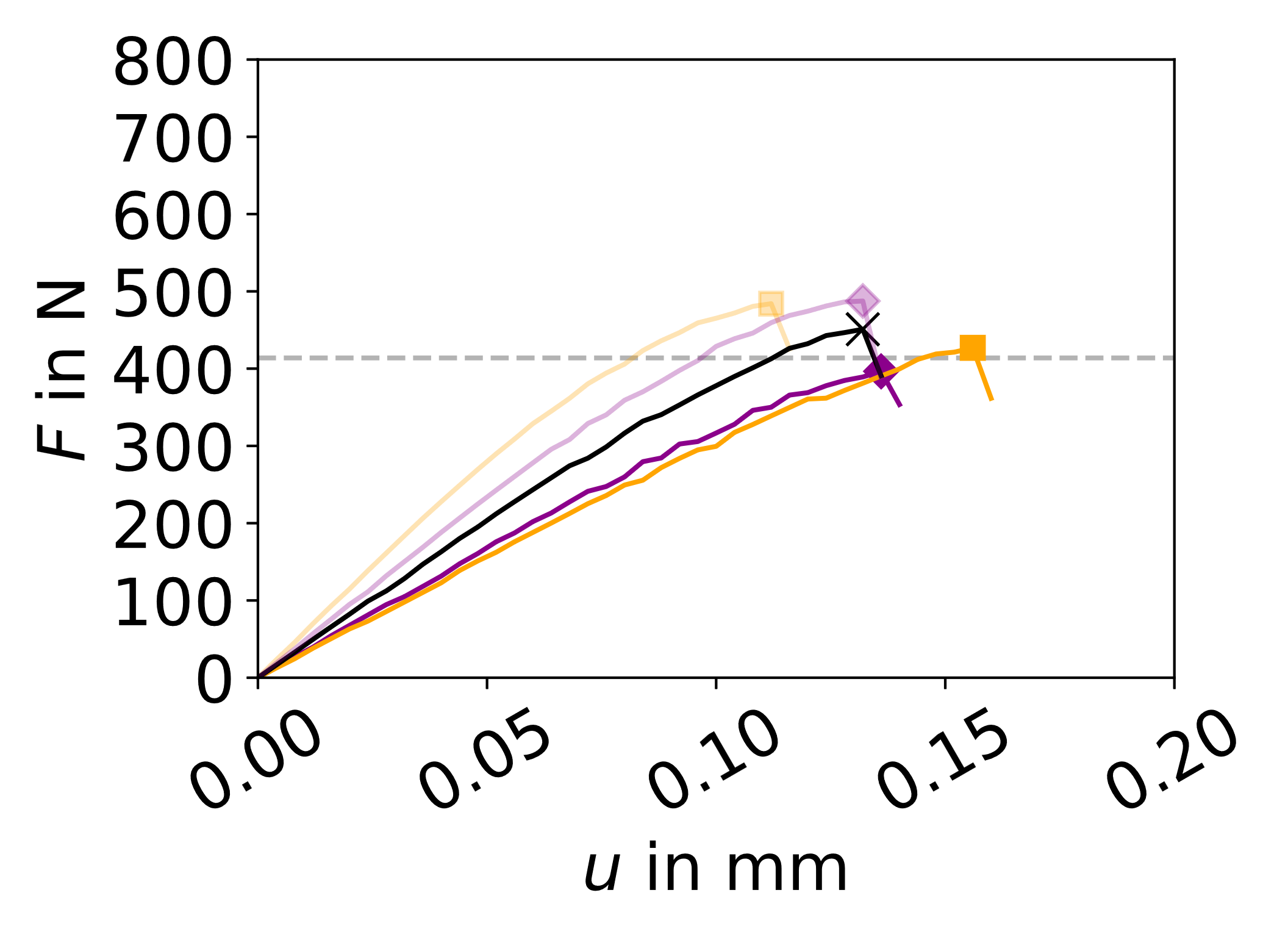 | 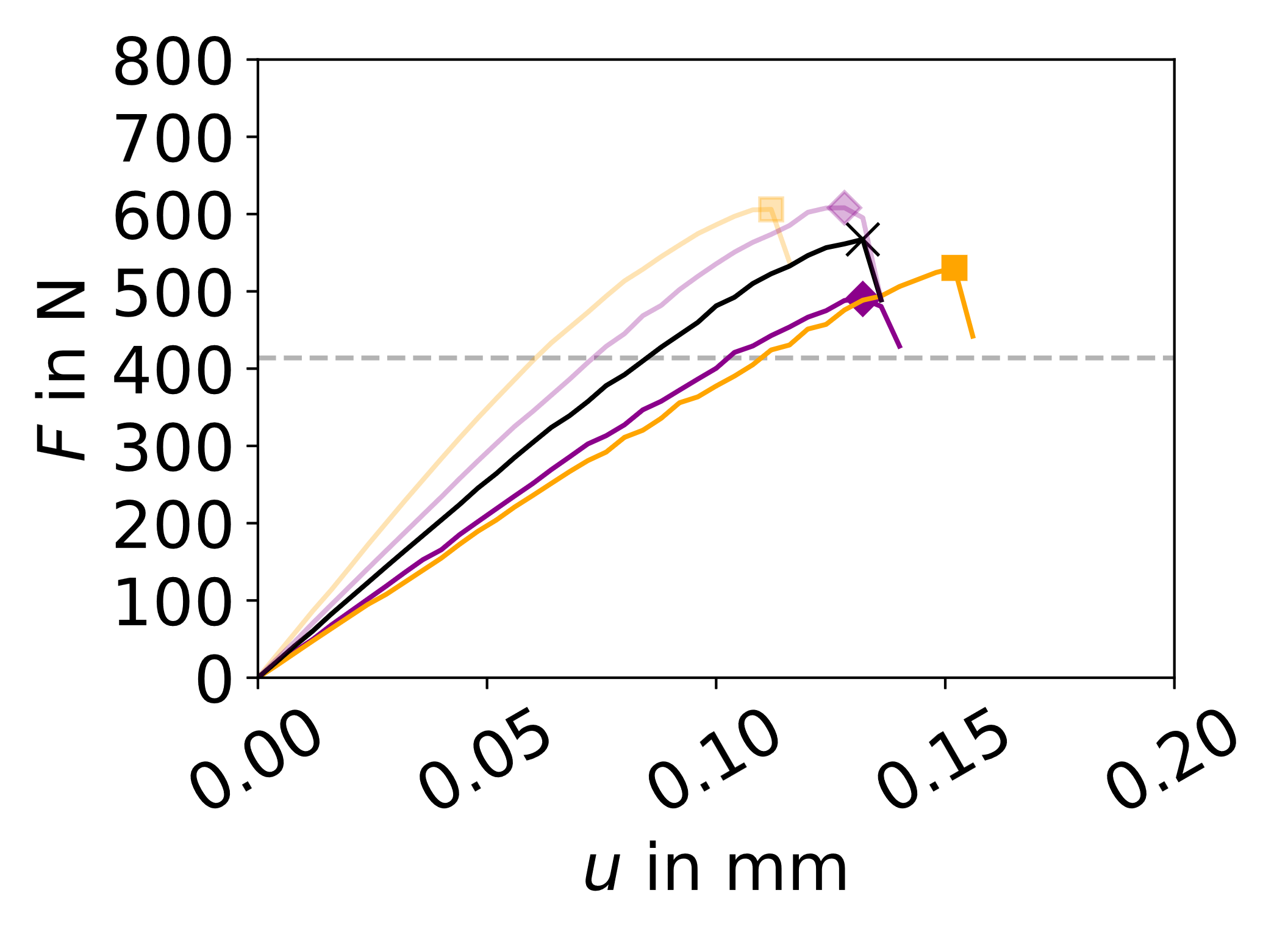 | | 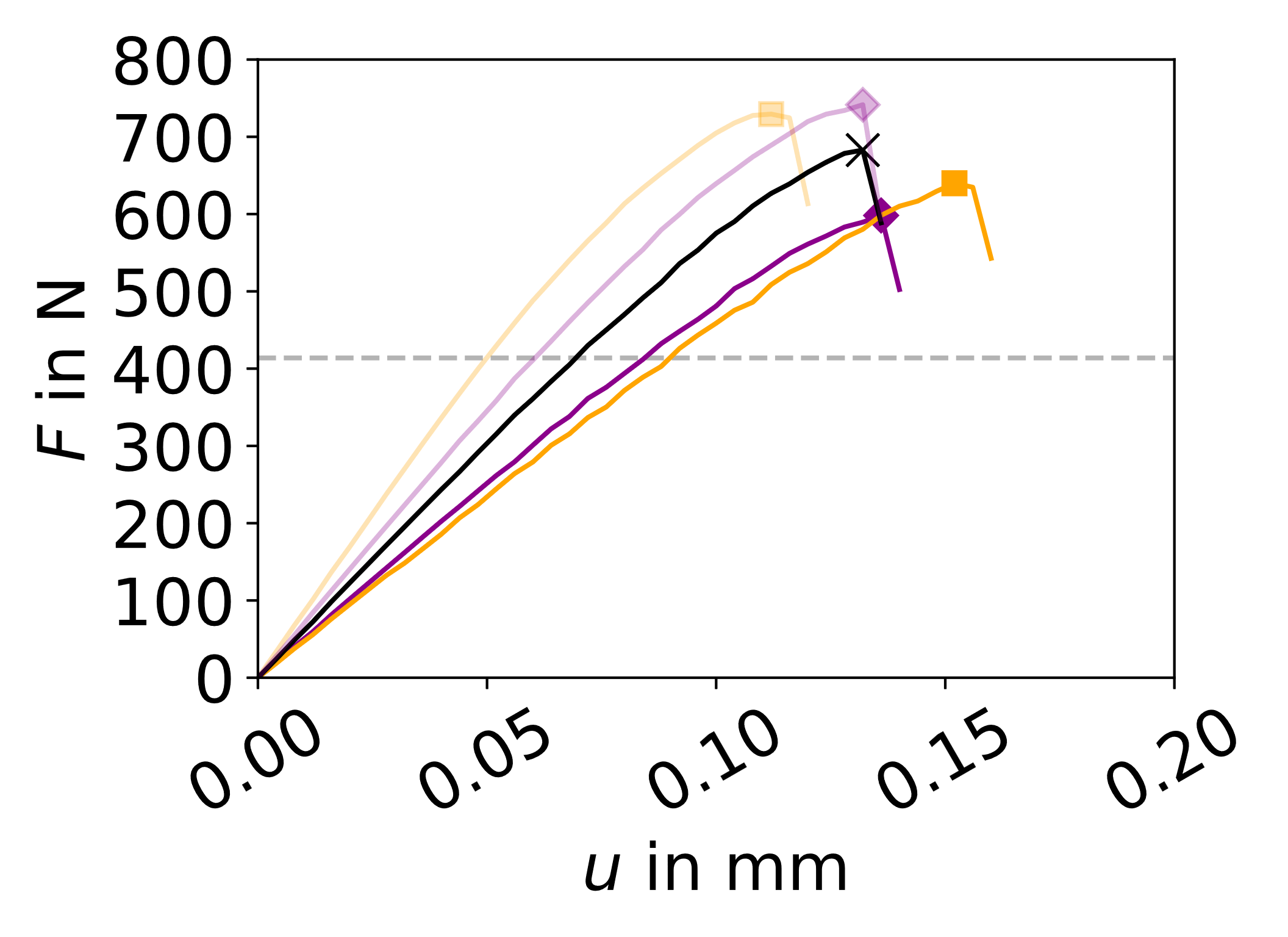 |
|  | ***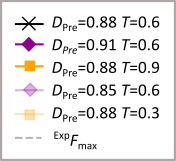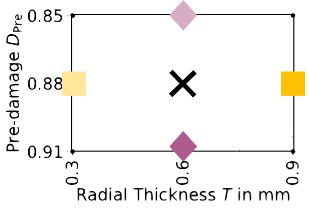*** | | | |
| **(C) Heat Maps** | 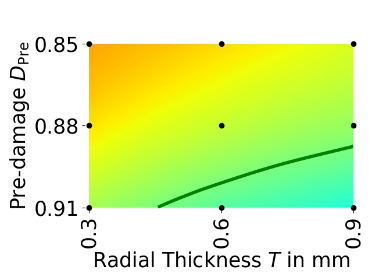 | 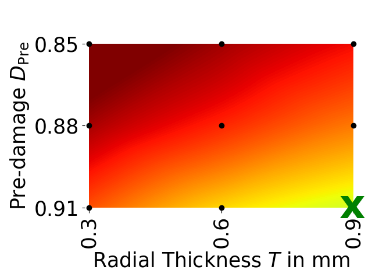 | | 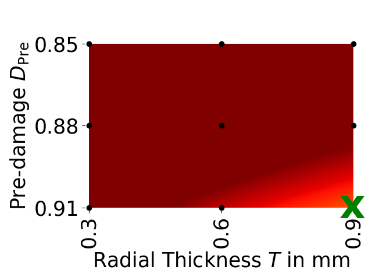 |
|  | 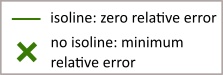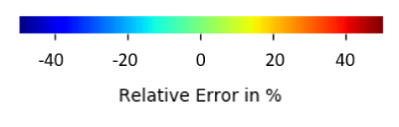 | | | |
| **Fig. S1.7:** Experimental force-displacement curve (**A**), simulated force displacement curves for different values of radial thickness *T* and pre-damage value *D*_Pre_ (**B**) and heat maps showing the relative error in maximum force (**C**) of specimen S7. Simulated force-displacement curves and heat maps are shown for three different elastic moduli of bone material *E*_red_=3.6GPa, *E*=4.6GPa, and *E*_inc_=5.6GPa. The simulated force-displacement curves (**B**) show a selection of five parameter combinations of *T* and *D*_Pre_. In the heat maps (**C**), green isolines mark the parameter combinations of pre-damage *D*_Pre_ and radial thickness of damage zone *T*, where the relative error in maximum force between simulation and experiment is zero. In case that no parameter combination can be found that leads to zero relative error, the parameter combination where the relative error is minimal is marked by a green cross. | | | | |

|  | **S8** | | | |
| --- | --- | --- | --- | --- |
| 1. **Experiment: Force - Displacement** | 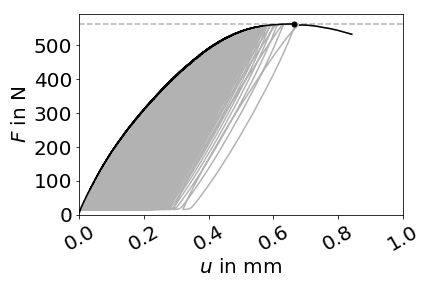 | | ***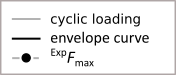*** | |
|  | ***E*_red_=3.6GPa** | ***E*=4.6GPa** | | ***E*_inc_=5.6GPa** |
| **(B) Simulation: Force - Displacement** | 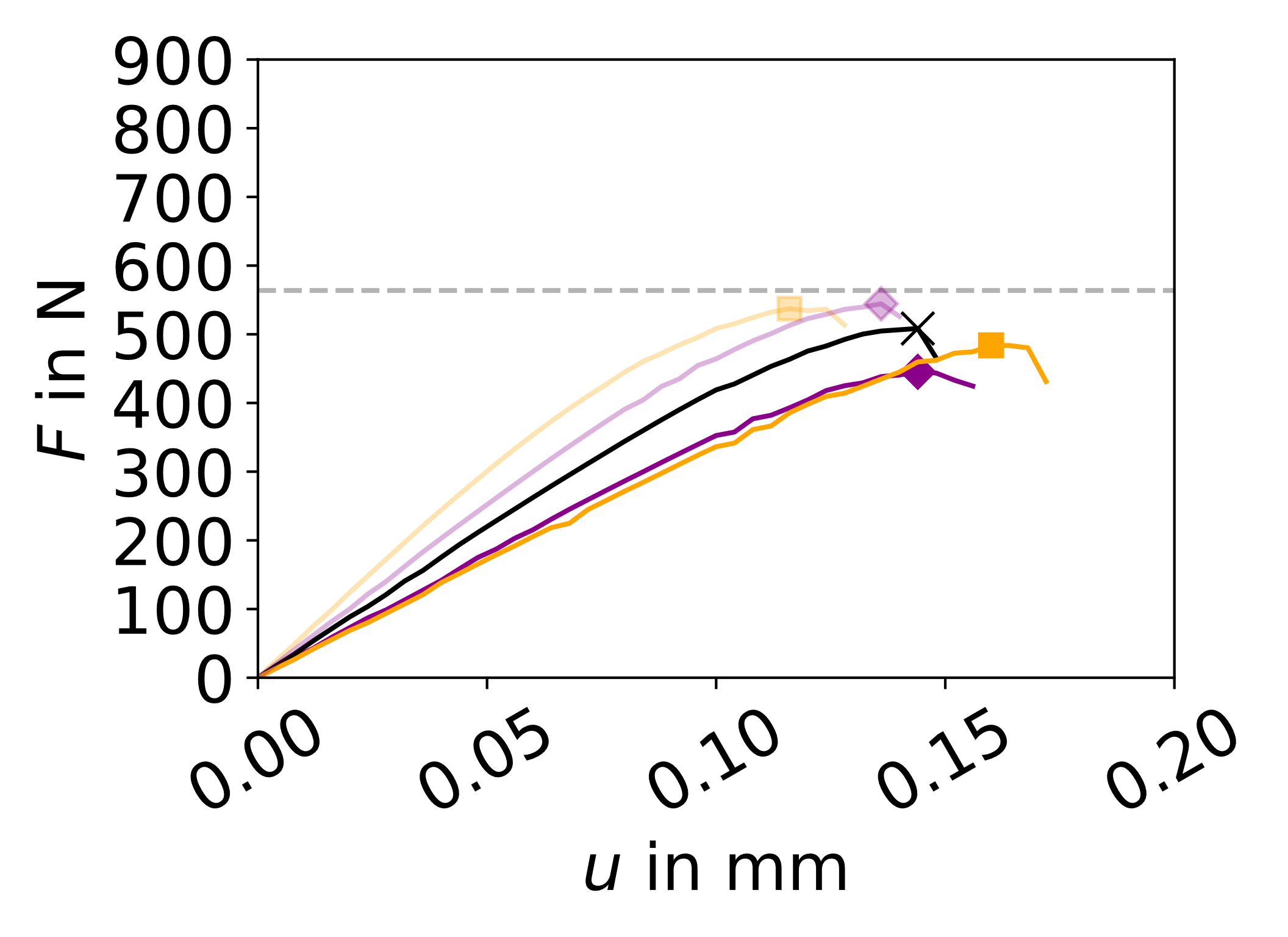 | 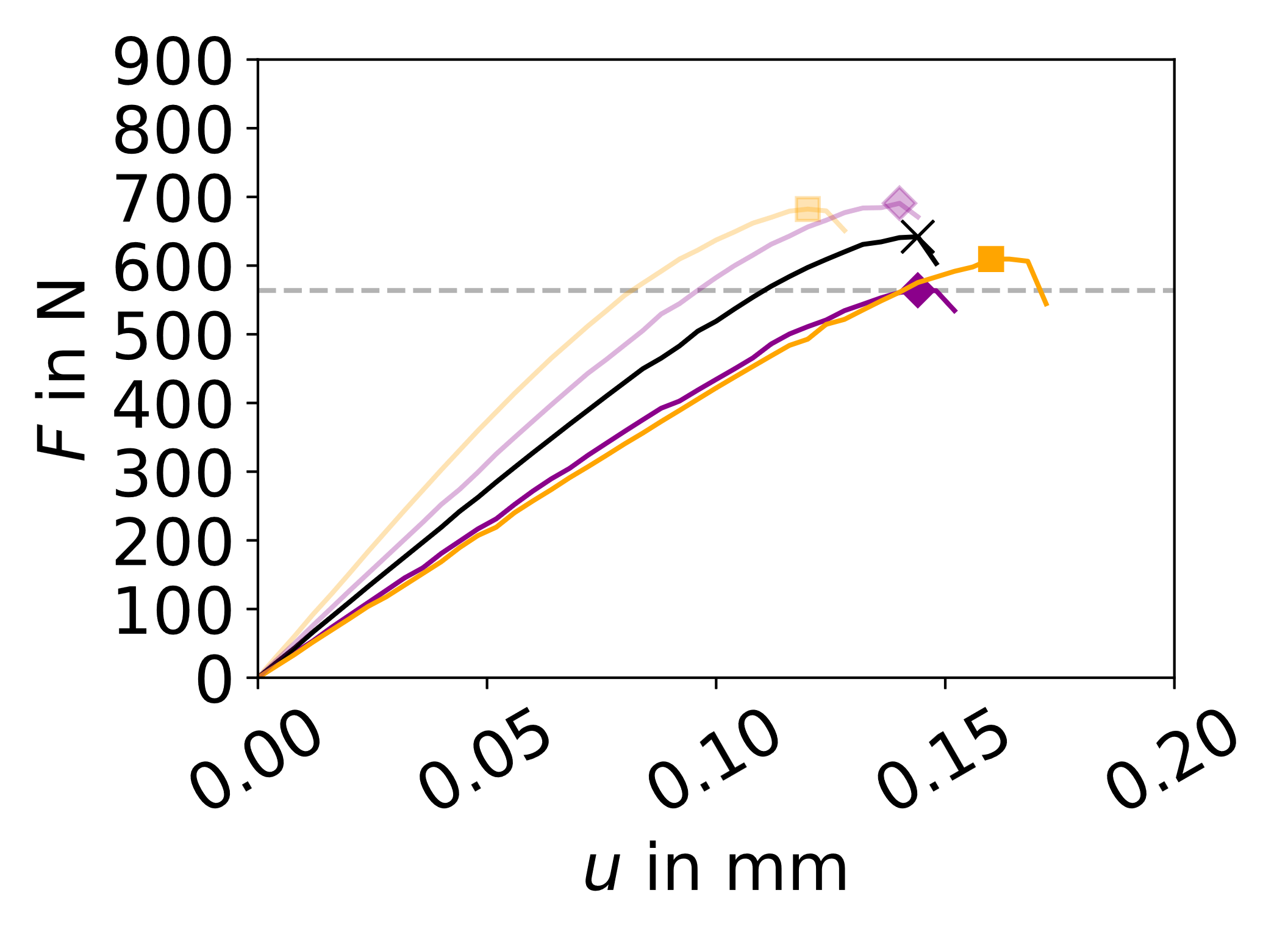 | | 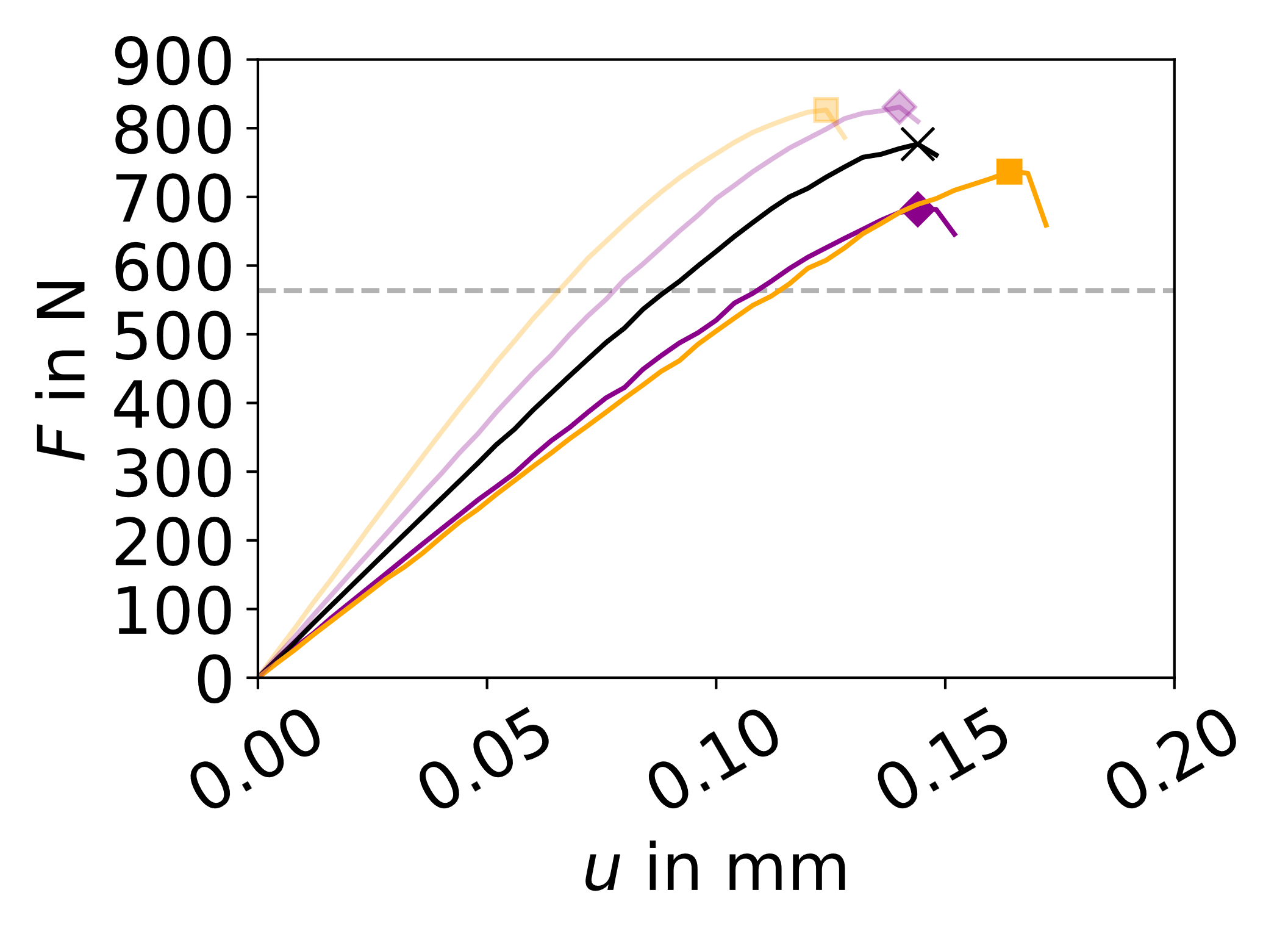 |
|  | ***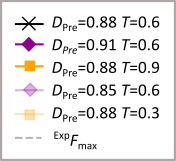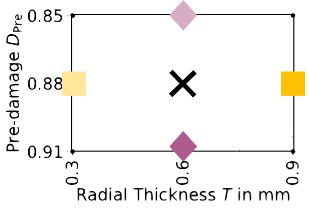*** | | | |
| **(C) Heat Maps** | 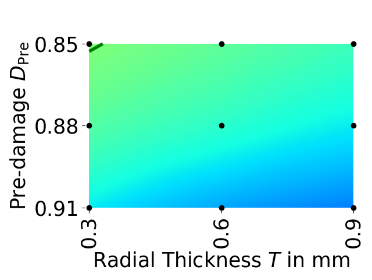 | 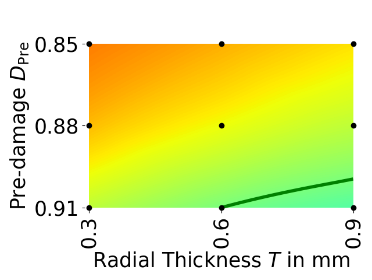 | | 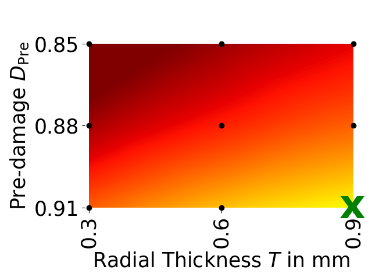 |
|  | 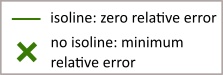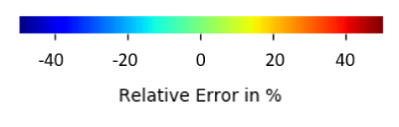 | | | |
| **Fig. S1.8:** Experimental force-displacement curve (**A**), simulated force displacement curves for different values of radial thickness *T* and pre-damage value *D*_Pre_ (**B**) and heat maps showing the relative error in maximum force (**C**) of specimen S8. Simulated force-displacement curves and heat maps are shown for three different elastic moduli of bone material *E*_red_=3.6GPa, *E*=4.6GPa, and *E*_inc_=5.6GPa. The simulated force-displacement curves (**B**) show a selection of five parameter combinations of *T* and *D*_Pre_. In the heat maps (**C**), green isolines mark the parameter combinations of pre-damage *D*_Pre_ and radial thickness of damage zone *T*, where the relative error in maximum force between simulation and experiment is zero. In case that no parameter combination can be found that leads to zero relative error, the parameter combination where the relative error is minimal is marked by a green cross. | | | | |

|  | **S9** | | | |
| --- | --- | --- | --- | --- |
| 1. **Experiment: Force - Displacement** | 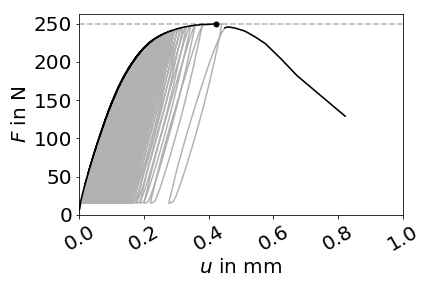 | | ***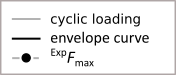*** | |
|  | ***E*_red_=3.6GPa** | ***E*=4.6GPa** | | ***E*_inc_=5.6GPa** |
| **(B) Simulation: Force - Displacement** | 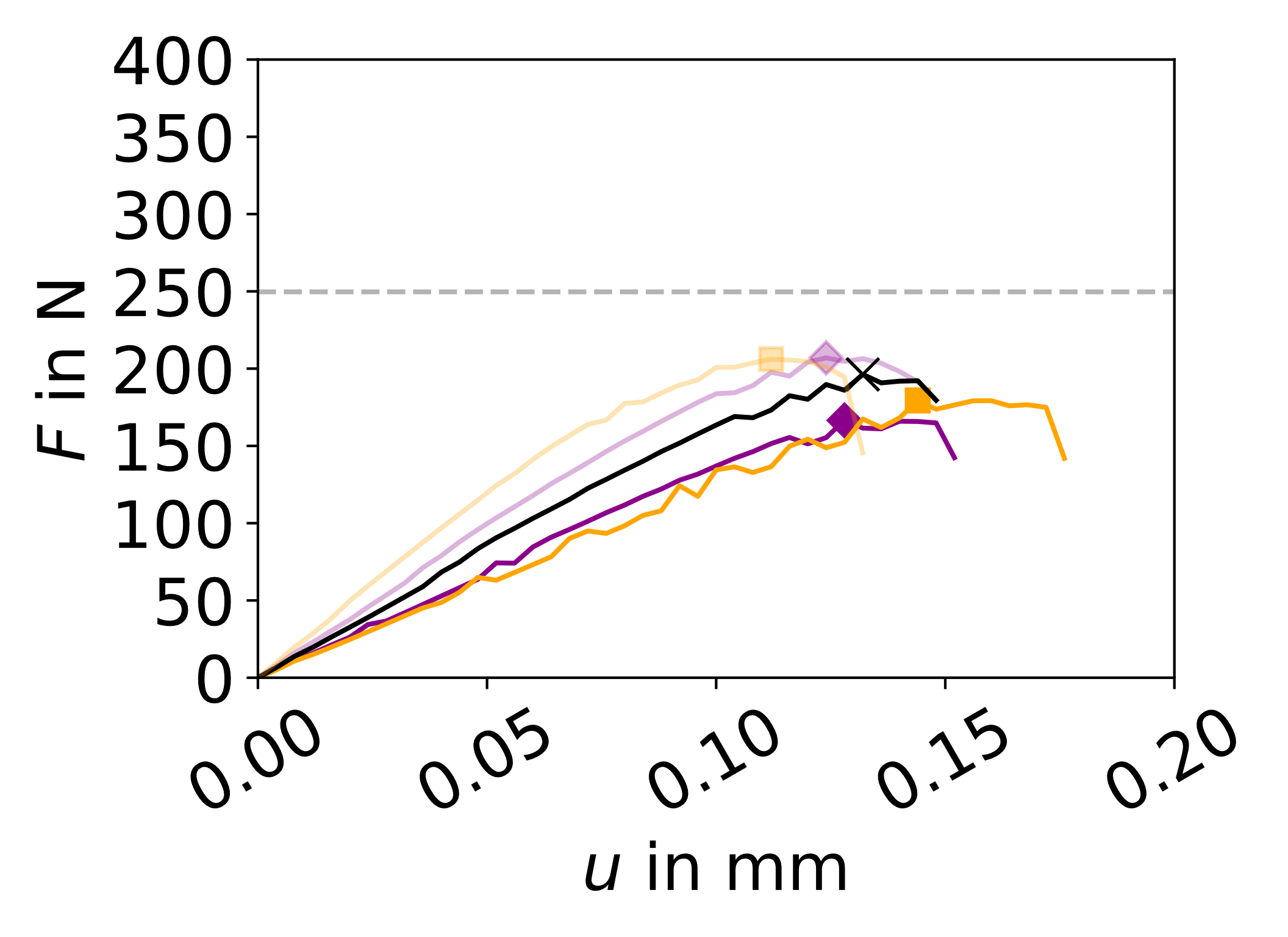 | 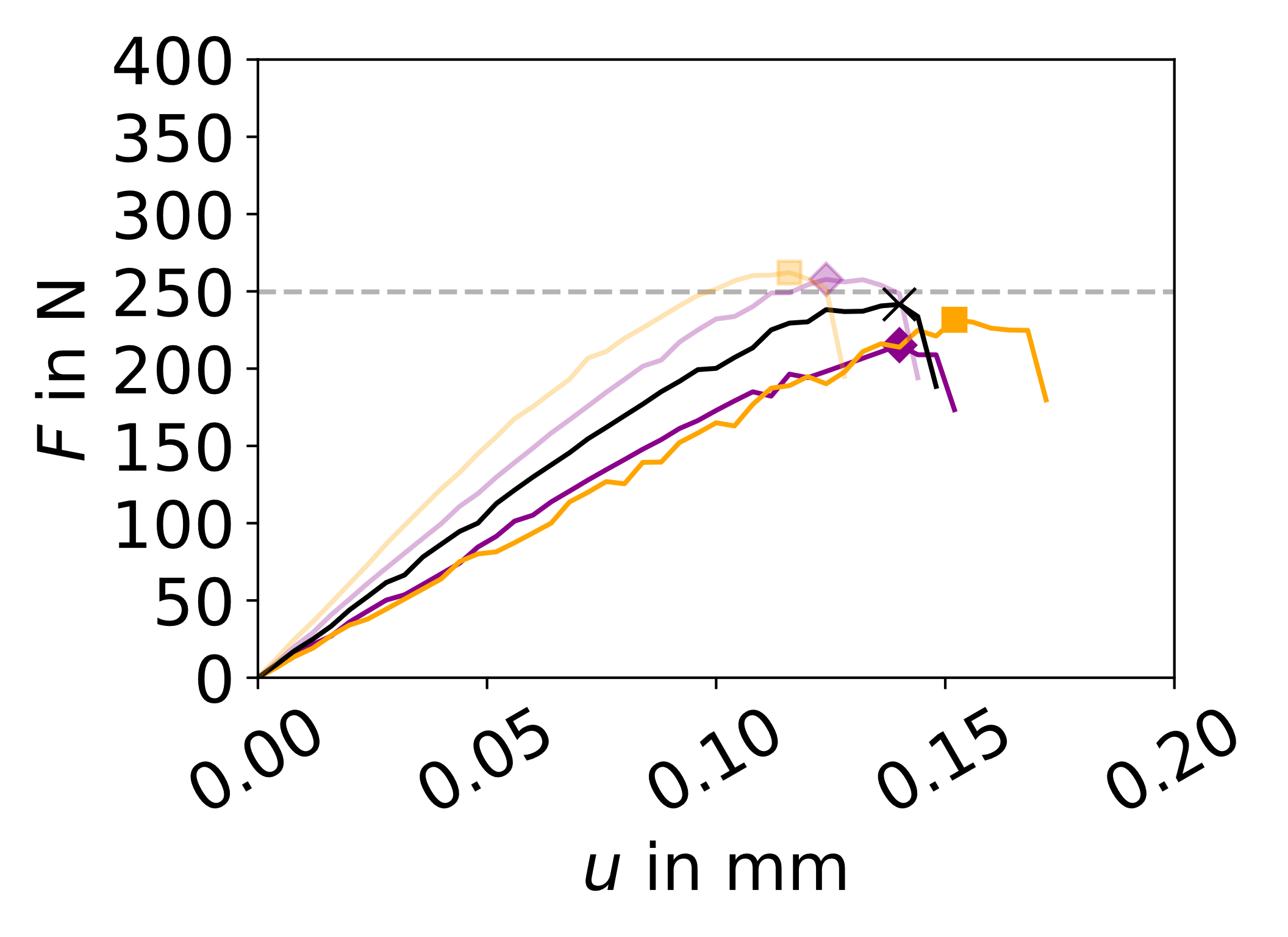 | |  |
|  |  | | | |
| **(C) Heat Maps** |  |  | |  |
|  |  | | | |
| **Fig. S1.9:** Experimental force-displacement curve (**A**), simulated force displacement curves for different values of radial thickness *T* and pre-damage value *D*_Pre_ (**B**) and heat maps showing the relative error in maximum force (**C**) of specimen S9. Simulated force-displacement curves and heat maps are shown for three different elastic moduli of bone material *E*_red_=3.6GPa, *E*=4.6GPa, and *E*_inc_=5.6GPa. The simulated force-displacement curves (**B**) show a selection of five parameter combinations of *T* and *D*_Pre_. In the heat maps (**C**), green isolines mark the parameter combinations of pre-damage *D*_Pre_ and radial thickness of damage zone *T*, where the relative error in maximum force between simulation and experiment is zero. In case that no parameter combination can be found that leads to zero relative error, the parameter combination where the relative error is minimal is marked by a green cross. | | | | |

**S2. Damage distribution at maximum force**

|  | ***E*=4.6GPa** | | | | | | |
| --- | --- | --- | --- | --- | --- | --- | --- |
|  | **FB** | **TED-M** |  | **TED-M+P**  **Min. *D*_Pre_** |  | **TED-M+P**  **Min. *T*** |  |
| **S1** |  |  |  |  |  |  |  |
| **S2** |  |  |  |  |  |  |  |
| **S3** |  |  |  |  |  |  |  |
| **S4** |  |  |  |  |  |  |  |
| **S5** |  |  |  |  |  |  |  |
| **S6** |  |  |  |  |  |  |  |
| **S7** |  |  |  |  |  |  |  |
| **S8** |  |  |  |  |  |  |  |
| **S9** |  |  |  |  |  |  |  |
| **S10** |  |  |  |  |  |  |  |
|  | **Fig. S2.1:** Damage at maximum force of all specimens for *E*=4.6GPa and for all interface and pre-damage combinations: a fully-bonded interface without pre-damage (FB) and a simplified interface method (Steiner et al., 2017 and Stefanek et al., 2024) without pre-damage (TED-M) and with pre-damage (TED-M+P). A displacement scaling factor of 5 was used, and optimal pre-damage parameters were selected based on two criteria: minimal pre-damage (Min. *D*_Pre_) and minimal radial thickness criteria (Min. *T*). For the TED-M+P, the color bar includes blue regions between the pre-damage value *D*_Pre_ in the damage zone and the critical damage *D*_c_. | | | | | | |

|  | ***E*_red_=3.6GPa** | | | | ***E*_inc_=5.6GPa** | |
| --- | --- | --- | --- | --- | --- | --- |
|  | **TED-M+P**  **Min. *D*_Pre_** |  | **TED-M+P**  **Min. *T*** |  | **TED-M+P** |  |
| **S1** |  |  |  |  |  |  |
| **S2** |  |  |  |  |  |  |
| **S3** |  |  |  |  |  |  |
| **S4** |  |  |  |  |  |  |
| **S5** |  |  |  |  |  |  |
| **S6** |  |  |  |  |  |  |
| **S7** |  |  |  |  |  |  |
| **S8** |  |  |  |  |  |  |
| **S9** |  |  |  |  |  |  |
| **S10** |  |  |  |  |  |  |
| **Fig. S2.2:** Damage at maximum force of all specimens for the two elastic moduli *E*_red_=3.6GPa and *E*_inc_=5.6GPa. All models included a simplified interface method (Steiner et al., 2017 and Stefanek et al., 2024) with pre-damage (TED-M+P). A displacement scaling factor of 5 was used, and optimal pre-damage parameters were selected based on two criteria: minimal pre-damage (Min. *D*_Pre_) and minimal radial thickness criteria (Min. *T*). For the TED-M+P, the color bar includes blue regions between the pre-damage value *D*_Pre_ in the damage zone and the critical damage *D*_c_. | | | | | | |
